# Comparative Analysis of Active Ingredients and Potential Bioactivities of Essential Oils from *Artemisia argyi* and *A. verlotorum*

**DOI:** 10.3390/molecules28093927

**Published:** 2023-05-06

**Authors:** Yun-Fen Wang, Yang Zheng, Yang Feng, Hao Chen, Shao-Xing Dai, Yifei Wang, Min Xu

**Affiliations:** 1 Center for Pharmaceutical Sciences, Faculty of Life Science and Technology, Kunming University of Science and Technology, Chenggong Campus, Kunming 650500, Chinafengy@kust.edu.cn (Y.F.); chenhao@kust.edu.cn (H.C.); 2 State Key Laboratory of Primate Biomedical Research, Institute of Primate Translational Medicine, Kunming University of Science and Technology, Kunming 650500, Chinadaisx@lpbr.cn (S.-X.D.); 3 Guangzhou Jinan Biomedicine Research and Development Center, Institute of Biomedicine, College of Life Science and Technology, Jinan University, Guangzhou 510632, China

**Keywords:** *Artemisia argyi*, *Artemisia verlotorum*, essential oils, chemical composition, networking pharmacology

## Abstract

*Artemisia argyi* H. Lév. and Vaniot is a variety of Chinese mugwort widely cultured in central China. *A. verlotorum* Lamotte, another variety of Chinese mugwort, has been used in the southern region of China since ancient times. Despite their similar uses in traditional medicine, little is known about the differences in their active ingredients and potential benefits. Herein, the chemical compositions of the essential oils (EOs) from both varieties were analyzed using chromatography-mass spectrometry (GC-MS). A series of databases, such as the Traditional Chinese Medicine Systems Pharmacology database (TCMSP), SuperPred database and R tool, were applied to build a networking of the EOs. Our results revealed significant differences in the chemical compositions of the two *Artemisia* EOs. However, we found that they shared similar ingredient–target–pathway networking with diverse bioactivities, such as neuroprotective, anti-cancer and anti-inflammatory. Furthermore, our protein connection networking analysis showed that transcription factor p65 (RELA), phosphatidylinositol 3-kinase regulatory subunit alpha (PIK3R1) and mitogen-activated protein kinase 1 (MAPK1) are crucial for the biological activity of *Artemisia* EOs. Our findings provided evidence for the use of *A. verlotorum* as Chinese mugwort in southern China.

## 1. Introduction

*Artemisia* spps. are grown in Asia, North America and Europe [1]. They are widely used as aromatic ethnic medicines in China, with their essential oils (EOs) being the major active ingredients frequently used in pharmaceuticals and cosmetics [2]. Due to the synergistic effects of a number of their active components, *Artemisia* EOs have pleiotropic bioactivity [3] and have attracted considerable attention for their biological diversities and bioactivities.

Chinese mugwort, made from the leaves of *A. argyi*, has a long history of use in China for controlling dysmenorrhea, bleeding and eczema [4,5]. The essential oils extracted from *A. argyi* (AAEOs) are one of the major active ingredients [6]. AAEOs have been shown to possess anti-oxidant [7,8], anti-inflammatory [9], anti-microbial [10], anti-fungal [11], anti-histamine [12], analgesic [13], anti-tumor and immunomodulatory effects [14]. It is noteworthy that *A. verlotorum*, a perennial plant widely distributed in the northern hemisphere [15], has similar folk medicinal uses as *A. argyi,* and is also widely used to make Chinese mugwort in southern China, especially in Guangdong province [16,17]. However, the chemical constituents and activities of the essential oils of *A. verlotorum* (AVEOs) are still unclear.

Herein, we analyzed the chemical ingredients of the EOs from different parts of *A. argyi* and *A. verlotorum* using gas chromatography-mass spectrometry (GC-MS) as the basis for potential activity exploration. We applied a networking pharmacology approach to study the interrelationships between the compounds, targets and related pathways for the EOs extracted from the leaves of *A. argyi* and *A. verlotorum*. The study involved a complex networking approach to investigate the interrelationships between the ingredients, target proteins and related signaling pathways of the EOs from both *Artemisia*. We further studied the statistical characteristics of the networking to explore the main active compounds and potential activities of the *Artemisia* EOs. Our results indicated that the ingredients of the EOs from the two *Artemisia* were different. The contents of monoterpenoids and sesquiterpenes could be used as markers to distinguish AAEOs from AVEOs. However, the networking pharmacology analysis suggested that the AAEOs and AVEOs have similar potential targets and pathways, indicating that they have similar biological activities. Our results provided evidence for the use of *A. verlotorum* as Chinese mugwort in southern China.

## 2. Results and Discussion

### 2.1. Chemical Compositions of EOs from Different parts of A. argyi and A. verlotorum

*A. argyi* and *A. verlotorum* have been used as Ay Tsao or Chinese mugwort in China [18]. *A. argyi* is known as northern Ay Tsao or northern Chinese mugwort, whereas *A. verlotorum* is called southern Chinese mugwort and is widely used by local people in southern China. Although many studies have reported the chemical constituents of *A. argyi*, few have reported on the chemical compositions of *A. verlotorum*. EOs are one of the major active ingredients of aromatic plants [19]. Previous studies have suggested that the yield of AAEOs was higher than AVEOs and the EOs of their leaves were higher than that of the aerial part [20]. However, systematic comparative studies of the constitutional differences between two Ay Tsao were unavailable. Herein, we analyzed the differences in the chemical compositions of EOs extracted from the whole grass of *A. argyi* (GAAEOs, YP-1), the leaves of *A. argyi* (LAAEOs, YP-2), the whole grass of *A. verlotorum* (GAVEOs, YP-3) and the leaves of *A. verlotorum* (LAVEOs, YP-4) using GC-MS (Table 1, Table S1, Figure S5).

**Figure 1:**
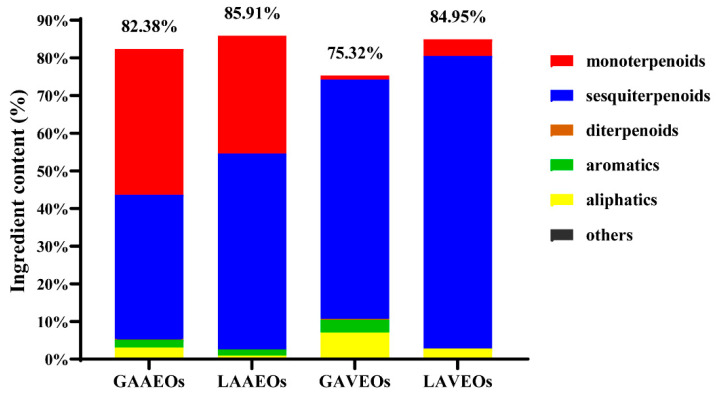
The chemical constituents of EOs from *Artemisia*.

A total of 132 volatile compounds of the *Artemisia* essential oils were identified using GC-MS, representing 75–86% of the total compounds (Table S1, Figure 1). The *Artemisia* EOs were abundant in terpenoids, including 70 sesquiterpenoids (45 sesquiterpenes, 18 sesquiterpenic alcohols, three sesquiterpenic ethers, two sesquiterpenic ketones and two sesquiterpenic esters), 37 monoterpenoids (nine monoterpenes, 15 monoterpenic alcohols, seven monoterpenic ketones, three monoterpenic esters, two monoterpenic ethers and one monoterpenic aldehyde) and three diterpenoids. Additionally, these EOs also contained five aromatics and 16 aliphatics. Among them, caryophyllene, caryophyllene oxide, neointermedeol and seven other ingredients were common components in the *Artemisia* essential oils. Diverse sesquiterpenoids accounted for the highest percentage of all *Artemisia* oils. The total contents of the sesquiterpeoids in the GAAEOs (38.42%), LAAEOs (52.01%), GAVEOs (63.56%) and LAVEOs (77.72%) were greater than 35%.

#### 2.1.1. Comparison and Analysis of the Chemical Composition of AAEOs and AVEOs

**Figure 2:**
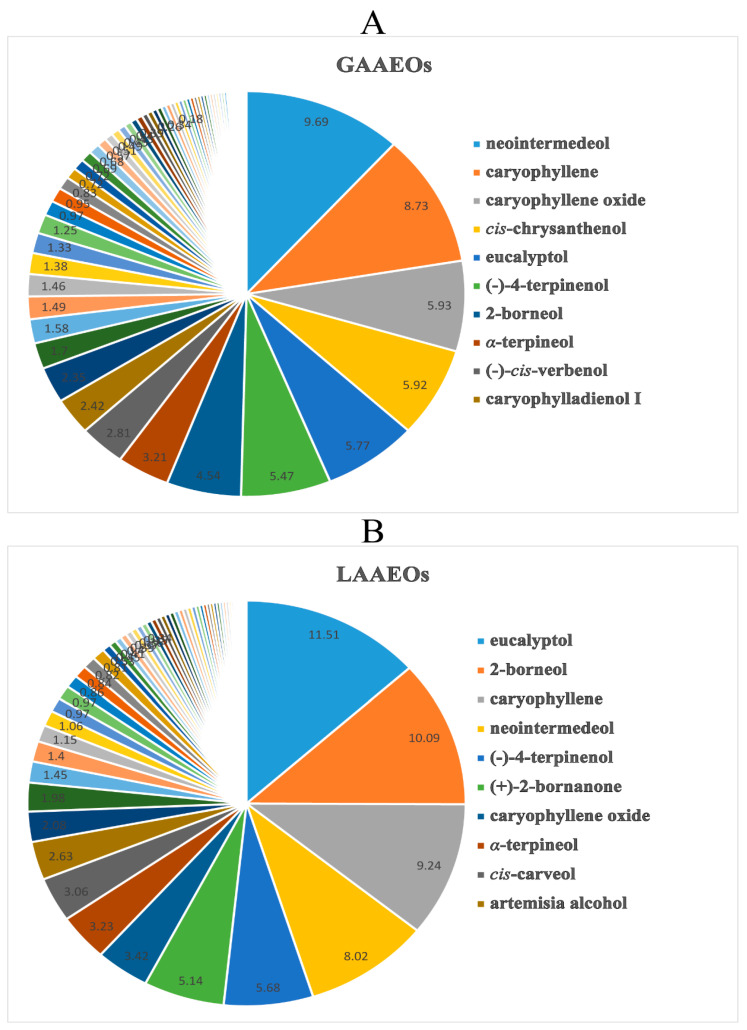
The chemical composition content of AAEOs. (**A**) The chemical composition content of essential oils whole grass from *A. argyi*. (**B**) The chemical composition content of essential oils leaves from *A. argyi*.

**Figure 3:**
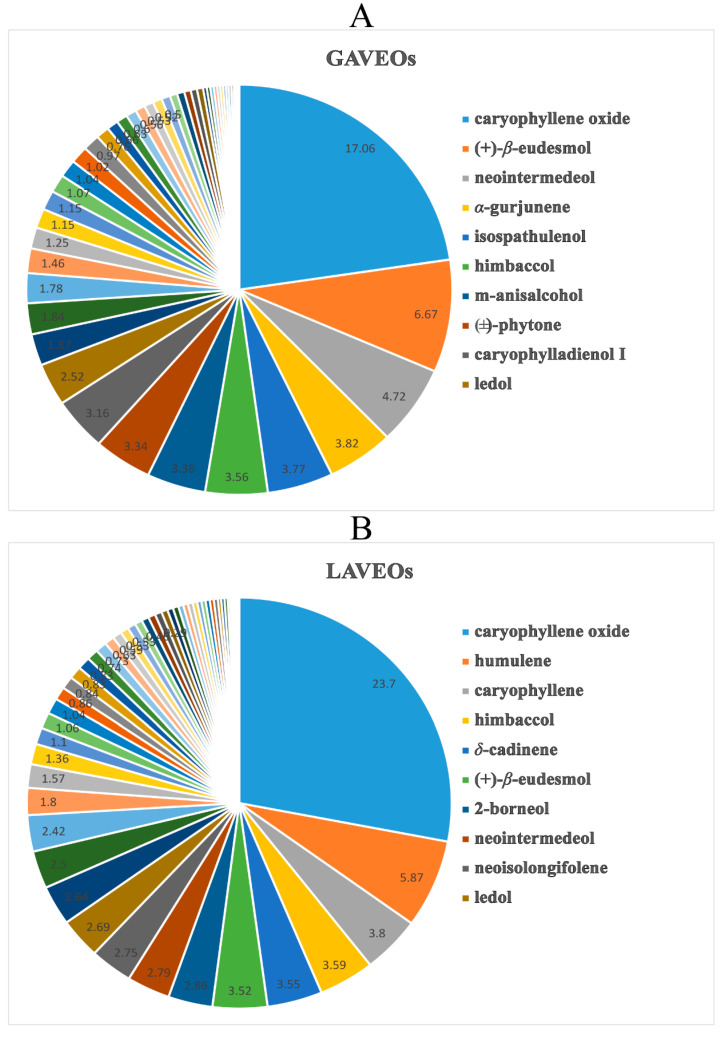
The chemical composition content of AVEOs. (**A**) The chemical composition content of essential oils whole grass from *A. verlotorum*. (**B**) The chemical composition content of essential oils leaves from *A. verlotorum*.

The chemical compositions of the Vs were more abundant than the AVEOs and differed significantly. Among them, neointermedeol and eucalyptol were the main ingredients of the AAEOs (Figure 2), while caryophyllene oxide was the primary component of the AVEOs (Figure 3). Furthermore, the content of monoterpenoids from the AAEOs (>30%) was higher than that of the AVEOs (<5%). However, sesquiterpenes had a higher proportion in the AVEOs (>60%) than in the AAEOs (<52%). Moreover, diterpenoids were only found in the whole grass of both AAEOs and AVEOs. The contents of monoterpenoids and sesquiterpenes may be used as markers to distinguish AAEOs from AVEOs.

#### 2.1.2. Comparison and Analysis of the EOs from Whole Grass and Leaves of A. Argyi

The *A. argyi* used in this study was collected in Henan province, China. For the EOs of the whole grass of *A. argyi* (GAAEOs, YP-1), 65 volatile compounds were identified. Among them, 22 monoterpenoids (38.71%), 27 sesquiterpenoids (38.42%), one diterpenoids (0.11%), four aromatics (2.03%) and 11 aliphatics (3.11%) were detected. Neointermedeol (9.69%) was the major compound, followed by caryophyllene (8.73%), caryophyllene oxide (5.93%), *cis*-chrysanthenol (5.92%) and eucalyptol (5.77%) (Figure 2A, Table S1). It is noteworthy that the chemical compositions of the GAAEOs showed less consistency across different locations. For instance, artemisia alcohol (44.52%) and yomogi alcohol (24.08%) were the principal components of GAAEOs originating from Korea reported in the literature [21]. The compounds and content of GAAEOs originating from different provinces in China also showed significant variation [3,18,22].

For the leaves of *A. argyi* (LAAEOs, YP-2) (Figure 2B, Table S1), we identified a total of 59 compounds, including 26 monoterpenoids (31.29%), 26 sesquiterpenoids (52.01%), three aromatic compounds (1.58%) and four fatty compounds (1.03%). Among them, eucalyptol (11.51%), 2-borneol (10.09%), (−)-4-*α*-terpinenol (5.68%) and (+)-2-bornanone (5.14%) were the major monoterpenes. Sesquiterpenoids accounted for a higher proportion and contain more abundant structural skeletons, of which caryophyllene (9.24%), neointermedeol (8.02%) and caryophyllene oxide (3.42%) were the principal components. Moreover, a few aromatic compounds, such as eugenol (0.84%), were observed, as well as aliphatics, such as 1-octen-3-ol (0.34%). The chemical composition of the LAAEOs was relatively consistent with previous reports [7,18,23]. Our results were consistent with a previous study on the constitute of LAAEOs, of which eucalyptol (9–33%) was considered a crucial constitute [18,24], as well as caryophyllene 2-borneol, (−)-4-*α*-terpinenol and humulene [9,25]. Additionally, artemisia alcohol (>30%) was reported to be a major component of LAAEOs collected from Korea [21]. However, artemisia alcohol had a lower content, of 2.64%, in LAAEOs from China. The cultivation location was considered to be an important factor in affecting the secondary metabolites of AAEOs. Some studies have established the GC-MS fingerprint of LAAEOs collected from Qichun county in Hubei province, identifying 23 common components as characteristic compositions [26]. However, only nine components, including eucalyptol, caryophyllene and borneol, were observed in our LAAEOs. Interestingly, all the principal components involved in the fingerprint, with the exception of thujone and alcanfor, were also detected in our EOs. Thujone, a neurotoxic substance commonly found in absinthe, was detected in multiple batches of the *A. argyi* samples [27]. Many important factors, such as the cultivation conditions, harvesting time, storage, extraction method and detecting conditions, influence the chemical constituents of the EOs of *A. argyi*.

The whole grasses of *A. argyi* were more chemically diverse than the leaves. The main chemical substances of the EOs from the two parts of *A. argyi* were similar, while the contents had some differences. There were 42 identical components, including neointermedeol, caryophyllene, caryophyllene oxide, etc., (Table S2) in both parts of the EOs. Neointermedeol and caryophyllene were the principal components of the whole grass (9.69% and 8.73%, respectively). They were also present at a high content in the leaves (8.02% and 9.24%, respectively). The main constituents of the LAAEOs were eucalyptol (11.51%) and 2-borneol (10.09%). However, the content of these two ingredients in the GAAEOs was only 5.77% and 4.54%, respectively. Notably, the LAAEOs had fewer compounds, but a higher content of major components than the GAAEOs, which may be one of the reasons why the leaves have been commonly used as medicinal herbs.

#### 2.1.3. Comparison and Analysis of the EOs from Whole grass and Leaves of *A. verlotorum*

The *A. verlotorum* used in this study was collected in Guangdong province, China. For the whole grass of *A. verlotorum* (GAVEOs, YP-3) (Figure 3A, Table S1), we detected 47 ingredients, including two monoterpenoids (1.05%), 37 sesquiterpenoids (63.56%), two diterpenoids, one aromatic compounds (3.36%), four fatty compounds (6.68%) and one phosphate (0.42%). Among them, caryophyllene oxide (17.06%), (+)-*β*-eudesmol (6.67%), neointermedeol (4.72%), *α*-gurjunene (3.82%) and isospathulenol (3.77%) were the major sesquiterpenoids. Similarly to the LAVEOs, the GAVEOs had a higher content and diverse structure of sesquiterpenes compared to monoterpenes. It is clear that the yield and constitutes of *Artemisia* oils may be influenced by the cultivation location, harvest time, vegetative cycle stage, extraction method and selection of plant parts, based on preliminary studies [18,28]. A comparison of the variance of terpenoid biosynthesis among different parts of *Artemisia* from the gene perspective suggested that there was a significant difference in the expression pattern of genes [29].

For the leaves of *A. verlotorum* (LAVEOs, YP-4) (Figure 3B, Table S1), we detected 55 volatile constituents. Among them, nine monoterpenoids (4.4%), 42 sesquiterpenoids (77.72%) and five aliphatics (2.83 %) were identified in the LAVEOs. Caryophyllene oxide was the major sesquiterpenoid in the LAVEOs, with more than 23% contents, followed by other sesquiterpenes, including humulene (5.87%), caryophyllene (3.80%) and *δ*-cadinene (3.55%). The sesquiterpenoids with significant structural diversity had a higher proportion (>65%) than those of monoterpenoids (<5%). The chemical compositions of the LAVEOs were completely different from those in previous reports. For example, germacrene D (23.6%) was the major component of AVEOs steam from Mauritius [30], which was not detected in our EOs. The major compounds *α*-thujone (47.0%), *β*-thujone (10.0%), eucalyptol (21.0%) and caryophyllene (3.4%) were identified from LAVEOs harvested in France [31]. However, eucalyptol, *α*-thujone (47.0%) and *β*-thujone were not detected in our EOs. Interestingly, the content of caryophyllene (3.8%) in our study was consistent with the report in this study [31]. 

The chemical composition of the GAVEOs was more abundant than that of the LAVEOs. Diterpenoids and aromatics were detected only in the whole grass. Interestingly, caryophyllene oxide (>17%) was the main substance in the EOs from different parts of *A. verlotorum*. Thirty common constituents were identified in the LAVEOs and GAVEOs, such as himbaccol, (+)-*β*-eudesmol, isospathulenol, etc. (Table S2).

### 2.2. Analysis of the Main Active Ingredients and the Potential Effect of EOs from A. argyi and A. verlotorum Using the Ingredient-Target-Pathway Networking 

**Figure 4:**
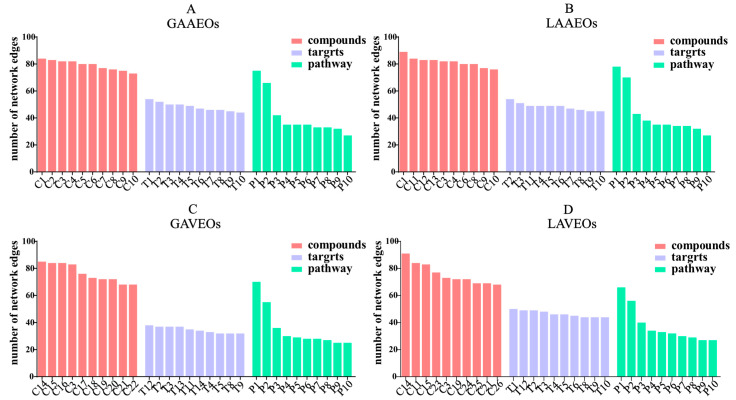
The top 10 components, targets and pathways of the number of networking edges (in Figure S2). (**A**) The number of networking edges from GAAEOs. (**B**) The number of networking edges from LAAEOs. (**C**) The number of networking edges from GAVEOs. (**D**) The number of networking edges from LAAEOs.

We analyzed the active ingredients, key targets and pathways of the different *Artemisia* EOs and created an “ingredient–target-pathway” map with a merge function. The networking was visualized using Cytoscape 3.9.1 software (Figure S2). The ingredients were represented by the purple V, the targets were represented by the yellow ellipse and the pathways were represented by the blue rectangle. The active ingredients were represented by the node, and the edge links represented the targets and the active ingredients. The high number of linkages demonstrated the importance of networking the active component or aim. Among them, the nodes of the GAAEOs (YP-1) included 55 components, 315 targets and 287 pathways, where neointermedeol had the highest number of networking edges. The nodes of the LAAEOs (YP-2) included 54 components, 329 targets and 287 pathways, where trimethylenenorbornane had the highest number of networking edges. The nodes of the GAVEOs (YP-3) included 39 components, 278 targets and 275 pathways, where pentamethylcyclopentadiene had the highest number of networking edges. The nodes of the LAVEOs (YP-4) included 50 components, 284 targets and 280 pathways, where 9-(1-methylethylidene)-1,5-cycloundecadiene had the highest number of networking edges. The results showed that the ten components with the highest connectivity in these EOs differed. In contrast, the targets of those ten components were quite similar. Moreover, they corresponded to exactly the same pathways (Figure 4, Table S3). The results suggest that although the components are different, they have similar biological activities. 

#### 2.2.1. Comparison and Analysis of Main Active Ingredients of AAEOs and AVEOs 

In order to focus on the possible active components, we investigated the drug-like characteristics of these molecules. The discovered compounds in the AAEOs and AVEOs were evaluated for their drug-like characteristics using Lipinski’s five guidelines. R was applied to analyze the correlation between the nodes. The nodes with a strong correlation were displayed with the same/similar color (Figure S3). The active ingredients contained in the top 50 scores are shown in Table 2, and the results showed that the ingredients with the highest scores among the four EOs were not the same. 

Methyleugenol, the highest scoring compound in the GAAEOs, was a phenylpropanoid used as a flavoring agent, a fragrance and an anesthetic in rodents [32,33]. However, the content of methyleugenol was only 0.17% in the GAAEOs, and it is unclear whether it is related to the activity of the EOs. In addition, no clear bioactivity studies have been reported for alloaromadendrene oxide (the highest-scoring compound in LAAEOs), *m*-anisalcohol (the highest-scoring compound in GAVEOs) and 9-(1-methylethylidene)-1,5-cycloundecadiene (the highest-scoring compound in LAVEOs).

**Figure 5:**
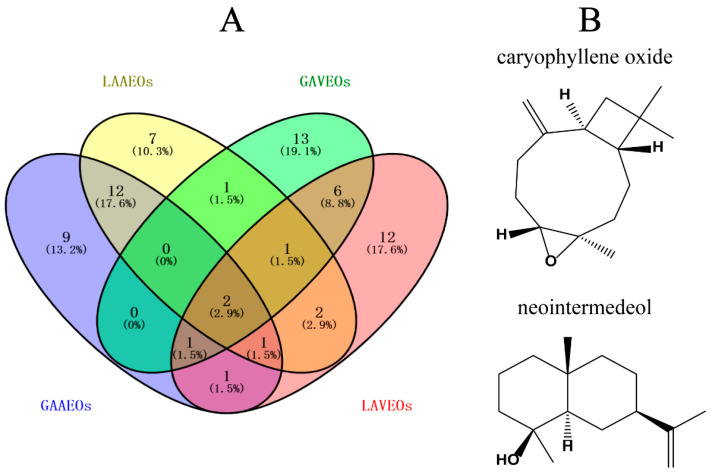
(**A**) Venny diagram of the number and ratio of EOs ingredients in Table 2. Venny analysis found that the EOs from different parts of two *Artemisia* share the same ingredients of caryophyllene oxide and neointermedeol. (**B**) Chemical structures of caryophyllene oxide and neointermedeol.

The caryophyllene oxide and neointermedeol were the common components of these four Eos, as shown in Table 2 using Venny analysis (Figure 5). Caryophyllene oxide has broad activities that have attracted much attention. It exhibited cytotoxicity against multiple cancer cells, including MG-63 (human osteosarcoma cells), HepG2 (human leukemia cancer cells), AGS (human lung cancer cells) and SNU-1 (human gastric cancer cell) [34,35]. Another study showed that caryophyllene oxide is a potent CNS depressant [36]. In addition, the CYP3A enzyme activity was markedly decreased by caryophyllene oxide, which could generally have an impact on the pharmacokinetics of the active compounds [37]. However, few studies have reported the biological activity of neointermedeol. 

#### 2.2.2. Comparison and Analysis of Key Proteins of AAEOs and AVEOs

Compounds show bioactivities by binding with particular proteins. [38]. Table 3 shows the target proteins included in the top 50 scores after clustering analysis by R. Among them, NFKB1 has the highest score in all of the essential oils. NFKB1 is a member of the NF-κB family and an important regulator of NF-κB activity in vivo. It has been shown that NFKB1 is closely associated with inflammation, aging and cancer in the body [39,40,41,42]. Thus, NFKB1 is one of the key targets of *Artemisia* essential oils with similar biological activity.

**Figure 6:**
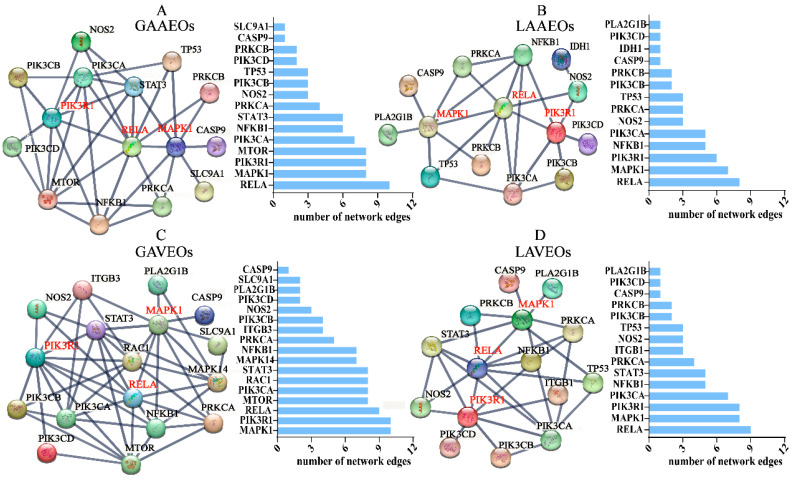
Protein-protein interaction (PPI) networking. Protein-protein interactions (set confidence level *p* > 0.9) targets from R cluster analysis. (**A**) Protein interaction network diagram of GAAEOs. (**B**) Protein interaction network diagram of LAAEOs. (**C**) Protein interaction network diagram of GAVEOs. (**D**) Protein interaction network diagram of LAVEOs.

A protein association network was constructed using STRING databases to screen the key target proteins with high interactions (Figure 6). The nodes encoded the networking of the target proteins. Furthermore, the protein–protein connection was defined as the connectivity degree. Genes with a high connectivity degree were defined as hub genes. The study found that RELA (transcription factor p65), PIK3R1 (phosphatidylinositol 3-kinase regulatory subunit α) and MAPK1 (mitogen-activated protein kinase 1) were the key targets and played a crucial role in various functions of the EOs from *Artemisia* in therapy. RELA (RELA Proto-Oncogene, NF-κB Subunit) is a pleiotropic transcription factor in practically all cell types. It is the endpoint of a series of signal transduction events that are sparked by various stimuli related to numerous biological processes, including tumorigenesis, differentiation, cell growth and apoptosis [43,44,45,46]. PIK3R1 functions as an adapter, mediating the association of the p110 catalytic unit to the plasma membrane. It binds to activated (phosphorylated) protein-Tyr kinases through its SH2 domain. This is required for the insulin-stimulated increase in glucose uptake and glycogen synthesis in insulin-sensitive tissues [47,48]. These targets are of great significance and deserve further investigation based on the relevant biological activity research before AAEOs, including their anti-inflammatory [9,49], neuroprotection [50], hypoglycemic and anti-oxidant [51], as well as anti-cancer [52,53] and other beneficial pharmacological, effects [18,54].

Additionally, the study found that the two MAPKs that are crucial to the MAPK/ERK cascade are MAPK1/ERK2 and MAPK3/ERK1. The regulation of transcription, translation and cytoskeletal rearrangements by the MAPK/ERK cascade regulates a variety of biological tasks, including cell growth, adhesion, survival and differentiation, depending on the cellular context [55,56]. Our results showed that although the ingredients were different from AVEOs and AAEOs, the targets of those ingredients were the same proteins. The results suggested that AVEOs and AAEOs have similar biological activities.

#### 2.2.3. GO Enrichment Analysis and KEGG Pathway Annotation

The biological processes, cellular components and molecular functions were the three major functional categories identified from the GO term enrichment analysis of the EOs. The top ten GO terms of each category are illustrated in Figure S4. The results showed that the cellular components had the most significantly enriched terms, with protein binding being the most significant cellular function among the four EOs. Protein binding can improve or impair a drug’s effectiveness [57].

**Figure 7:**
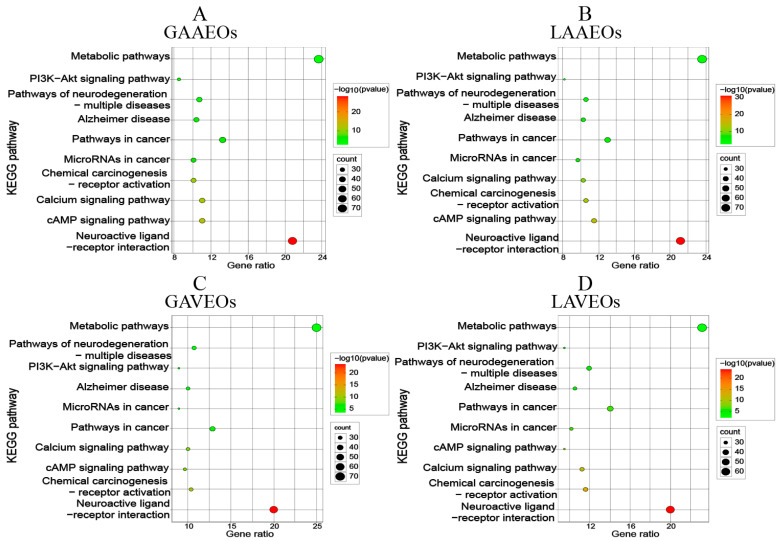
Bubble diagram of KEGG enrichment analysis of EOs from *Artemisia*. (**A**) KEGG pathway of GAAEOs. (**B**) KEGG pathway of LAAEOs. (**C**) KEGG pathway of GAVEOs. (**D**) KEGG pathway of LAVEOs. *Y*-axis label represents the pathway, and *X*-axis label represents the gene ratio. Each bubble represents an enriched function, and the size of the bubble represents the number of genes enriched in the pathway. The bubble is colored according to its −log (*p* value).

To identify the pathways and targets that were involved directly, the pathway information related to the targets was obtained through KEGG analysis. Figure 7 shows that the associated pathways mainly included metabolic pathways, the neuroactive ligand–receptor interaction and pathways in cancer. Substantial experimental evidence showed that AAEOs had good activities in neuroprotection [50], anti-inflammatory, analgesic and anti-tumor effects [18,54], which were consistent with the prediction of networking pharmacology. Further analysis based on GO and KEGG showed that the enrichment results of the AAEOs and AVEOs were highly consistent, indicating that AVEOs and AAEOs might have the same action pathway and similar pharmacological activities.

#### 2.2.4. Network Analysis of the Unique Components of the Four *Artemisia* Essential Oils

**Figure 8:**
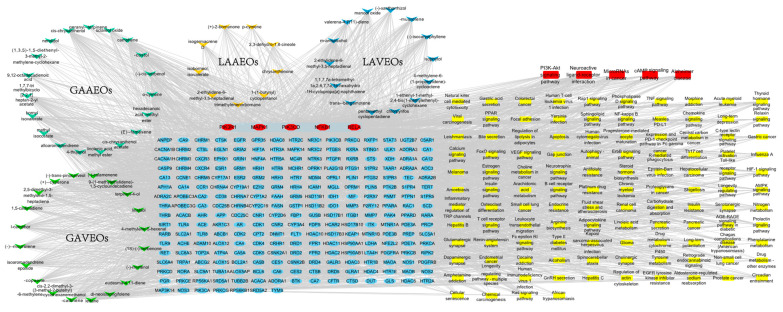
Network diagram between the unique components of *Artemisia* EOs (GAAEOs, LAAEOs, GAVEOs, LAVEOs) and targets, and pathways. Red nodes represent the targets and pathways with the highest degree of association.

Furthermore, we constructed a network between the unique composition of *Artemisia* essential oils (GAAEOs, LAAEOs, GAVEOs, LAVEOs) and its targets and pathways **(**Figure 8). The key targets and pathways of the top five (red nodes) revealed by the enrichment results were highly consistent with the results of the GO and KEGG analyses (Figure 6 and Figure 7), further illustrating that the two different *Artemisia* essential oils have similar pharmacological activities despite significantly differing in their compositions. In addition, the key targets have been proved to mediate the relevant signaling pathways to exert immunomodulatory, anti-inflammatory and neuroprotective activities, etc. [18,58,59,60].

### 2.3. In Vitro and In Vivo Toxicity of Artemisia Essential Oils

**Figure 9:**
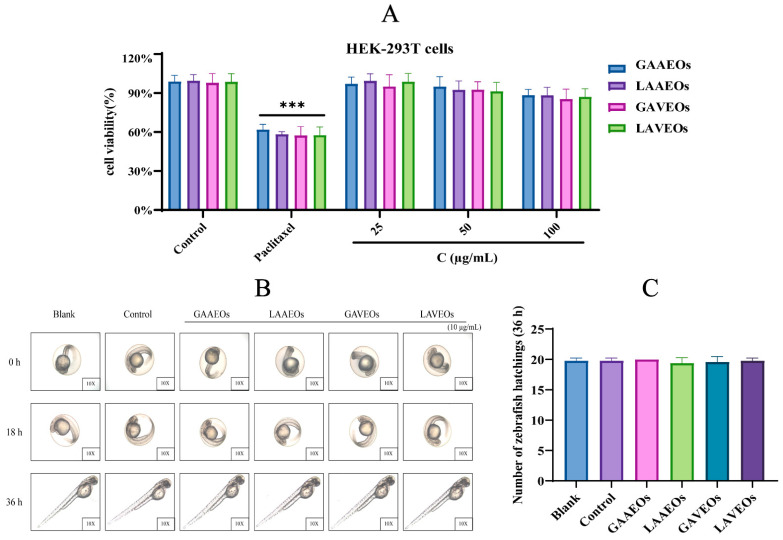
Toxicity test results for *Artemisia* essential oils. (**A**) Effect of *Artemisia* essential oils on the viability of HepG2 cells. (*** *p* < 0.001 positive group vs. control group). (**B**) Effect of *Artemisia* essential oils on the viability of A549 cells. (**C**) Effects of *Artemisia* essential oils (10 µg/mL) on the development and growth of zebrafish. All data were presented as means ± SD (n = 5), calculated by student *t* test.

As a processing of Chinese materia medica widely used in clinic, the toxicological evaluation of the *Artemisia* essential oils was particularly important. In order to better ensure the drug safety, it can be assumed that the biological activity of *Artemisia* essential oils was assessed through in vitro and in vivo toxicity tests. The results of the cellular level assay showed that 100 μg/mL of *Artemisia* essential oils did not show cytotoxic activity against both HEK-293T cell lines treated for 24 h (Figure 9A,B). Furthermore, the in vivo assay in zebrafish showed that 10 μg/mL of *Artemisia* essential oils did not affect the growth and survival of zebrafish (Figure 9C). This study presents the first systematic assessment of the toxicity of *Artemisia* essential oils using an in vitro human normal cell line (HEK-293T cells) assay in combination with an in vivo zebrafish assay. In this regard, *Artemisia* essential oils are highly safe.

## 3. Conclusions

Aromatic chemical components from folkloric medicinal plants, such as EOs, have been claimed to be useful in treating or preventing a variety of illnesses [61]. The fragrant medicinal plants of the *Artemisia* species have complicated locations and origins [1]. Although the compounds and activity of *A. argyi* have been investigated, the mechanism by which these components act on human health at the cell level has remained largely unknown. Few studies have focused on the biological activity of the essential oils of *A. verlotorum*. 

Herein, we analyzed the compositional differences and potential bioactivities of EOs from two Chinese mugworts. The results showed that the chemical composition of the EOs from *A. argyi* and *A. verlotorum* were quite different. However, networking pharmacology and R cluster analysis studies showed that they share similar key target proteins, as well as highly consistent protein interactions and signaling pathways. This indicated that these two *Artemisia* essential oils could be substituted for each other in aromatic therapy. Our study provides evidence to better understand the development and application of *A. argyi* and *A. verlotorum* in Chinese traditional medicine and lays a foundation for the clinical safety and scientific medication of *Artemisia* essential oils.

## 4. Materials and Methods

### 4.1. Plant Materials and Reagent

The *A. argyi* H. Lév. and Vaniot and *A. verlotorum* Lamotte were derived from different species of the same genus and have extremely similar morphological characteristics. These samples were (n = 3) collected from Nanyang, Henan Province, and Luofo mountain, Guangdong Province (specific medicinal plant cultivation sites) [62,63], People’s Republic of China, respectively, and were collected strictly according to the medicinal age (1 year) and time of harvest. Both plants were identified by Prof. Chong-Ren Yang. The materials were dried in the shade at 25 °C until the humidity was lower than 5%, and stored in the refrigerator at 4 °C. Voucher specimens (more than 100 mg) were deposited at the Center for Pharmaceutical Sciences, Faculty of Life Science and Technology, Kunming University of Science and Technology, Kunming, P. R. China.

### 4.2. Extraction of EOs A. verlotorum

Steam distillation was used to extract the EOs from different parts of the whole grass and leaves of *A. argyi* (YP-1 and YP-2) and *A. verlotorum* (YP-3 and YP-4). The dried *Artemisia* (100 g), in a round bottom flask, was added to distilled water (1 L). The extractions of Eos, in detail, followed previous studies [64]. Detailed information of all the EOs is presented in Table 1.

### 4.3. GC-MS Analysis

GC-MS was used to analyze the compounds of AAEOs and AVEOs. The methods, in detail, followed previous studies [64].

### 4.4. Chemical Ingredients Database Building of EOs

The discovered chemicals in the EOs were screened for drug-like characteristics using Lipinski’s five rules [65]. The Traditional Chinese Medicine Systems Pharmacology database (TCMSP) (http://tcmspw.com/tcmsp.php, 18 February 2022) [66] and PubChem database (https://pubchem.ncbi.nlm.nih.gov/, 18 February 2022) were used to assess these crucial pharmaceutical features.

### 4.5. Collection of Target Proteins and Pathways of the EOs

Potential targets of the compounds in the AAEOs and AVEOs were collected via networking databases (probability > 0.7), including SuperPred (https://prediction.charite.de, 20 February 2022), SwissTargetPrediction (http://www.swisstargetprediction.ch/, 20 February 2022) and TargetNet (http://targetnet.scbdd.com/, 20 February 2022). The ingredients’ absent target proteins were eliminated. In addition, the information on all the collected proteins was made uniform using Uniprot (http://www.uniprot.org/, 3 March 2022) [67]. 

Using the Kyoto Encyclopedia of Genes and Genomes (KEGG) pathway database (http://www.kegg.jp/kegg/, 4 March 2022) and the Database for Annotation, Visualization and Integrated Discovery (DAVID) (https://david.ncifcrf.gov/summary.jsp, 4 March 2022), the analysis of pathways was carried out on the chosen targets. The database is an encyclopedia of genes and genomes and includes information on signal transduction, cellular biology and homologous conservative route [68]. The study uses Human sapiens as model species.

### 4.6. Networking Construction

Cytoscape 3.9.1 was used to analyze and visualize the data gathered to create complicated networks [69]. In this network, nodes stood in for components, targets or pathways, while edges denoted their connections. Subsequently, the tight lines and complexities of the connections between important chemical components, targets and pathways were considered to explore the underlying mechanism of action. Therefore, cluster analysis of the relevant collected information was performed by R (cluster_louvain). The related ingredients, targets and pathways information resulted in a data set that was converted to an igraph graph using the “igraph” software package. A function of graph_from_incidence_matrix creates a bipartite igraph graph from the incidence matrix of the data for targets and pathways. Based on the bipartite igraph graph, a function of igraph_cluster_louvain implements the multi-level modularity optimization algorithm for finding community structure, and different communities were marked in different colors by a R_rainbow, while their relationships were analyzed using the igraph_deg function [70].

Based on the top targets in the R processing results, protein–protein interaction networking analysis (PPI) was also carried out to assess the targets. The association between the targets was evaluated as follows. PPI: To show how the target proteins interact, the target proteins were uploaded to the STRING databases platform (http://string-db.org, 6 April 2022). In this study, we eliminated the isolated targets and constructed a PPI networking by screening them with a confidence score > 0.90.

### 4.7. Gene Ontology and Pathway Enrichment Analysis

Gene ontology (GO) enrichment analysis was performed on the candidate targets using the online tool DAVID and KEGG, as well as KEGG pathway annotation. *p* values < 0.05 were considered statistically significant.

### 4.8. Toxicity Analysis

Cytotoxicity assay: The toxicity of *Artemisia* essential oils on HEK-293T cells (human embryonic kidney 293 cells, were obtained from the Kunming Cell Bank of Chinese Academic of Sciences) was detected using the MTT method, consistent with the previous study [71]. Cells were treated with *Artemisia* essential oils (25, 50, 100 μg/mL) for 24 h. The cells incubated with 3 µM paclitaxel (PTX) (Adamas Reagent Co., Ltd., 48803A, Shanghai, China) was used as the positive control (paclitaxel was drugs widely used clinically for the treatment of cancer).

Zebrafish toxicity assay: Healthy 24 h post-fertilization (hpf) embryos (transparent fertilized eggs) were randomly transferred to different concentrations of methanol extract of *Artemisia* essential oils (10 μg/mL) in the sterile 12 well plate. Each group was provided with 20 embryos (repeated experiment, n = 5). The development and morphological changes of the embryos after drug exposure were observed, photographed and recorded using an inverted optical microscope at 18 and 36 hpf, and recorded the hatching rate of the embryos at 36 hpf.

### 4.9. Statistical Analysis

All the data were statistically analyzed using the GraphPad Prism 9 software, using a two-tailed Student’s t-test. *p* values < 0.05 were considered statistically significant.

## Figures and Tables

**Table 1:** Yields of the EOs from different parts of two *Artemisia* spps.

No.	Latin Name	Plant Parts	Abbreviation	Appearance of Essential Oils	Collecting Locations	Yield (*v*/*w*, %)
YP-1	*A. argyi*	whole grass	GAAEOs	blue	Nanyang, Henan	0.15
YP-2	*A. argyi*	leaves	LAAEOs	blue	Nanyang, Henan	0.34
YP-3	*A. verlotorum*	whole grass	GAVEOs	yellow-green	Luofo mountain, Guangdong	0.015
YP-4	*A. verlotorum*	leaves	LAVEOs	yellow-green	Luofo mountain, Guangdong	0.032

**Table 2:** Ingredients included in the top 50 of the score by R cluster analysis.

GAAEOs	NO ^a^	Scores ^b^	LAAEOs	NO ^a^	Scores ^b^	GAVEOs	NO ^a^	Scores ^b^	LAVEOs	NO ^a^	Scores ^b^
methyleugenol	C37	8462.68	alloaromadendrene oxide	C44	8986.16	m-anisalcohol	C34	11053.29	9-(1-methylethylidene)-1,5-cycloundecadiene	C14	6343.27
1-octen-3-ol	C9	7899.98	methyleugenol	C26	8884.00	alloaromadendrene oxide	C32	8097.39	4-methylene-5-hexenal	C13	5064.08
*o*-cymene	C40	7509.06	1-octen-3-ol	C6	8458.30	pentamethylcyclopentadiene	C35	7295.15	*β*-elemene	C46	5060.33
chamazulene	C22	6276.07	chamazulene	C15	6509.30	*β*-elemene	C29	6874.15	*β*-calacorene	C44	4812.89
eugenol	C32	5706.63	*p*-cymene	C49	6502.40	*β*-calacorene	C21	5972.17	(+)-*α*-calacorene	C5	4325.45
bornyl isovalerate	C16	4615.64	trimethylenenorbornane	C50	5701.88	neointermedeol	C17	5612.14	bornyl acetate	C16	4218.15
*cis*-carveol	C23	4358.01	eugenol	C22	5568.67	chlorpyrifos	C33	5080.56	caryophyllene oxide	C20	4076.81
junenol	C35	4222.82	*β*-elemene	C53	5117.62	(+)-*α*-calacorene	C1	4997.34	2,5-dimethyl-3-methylene-1,5-heptadiene	C11	4066.86
neointermedeol	C38	3900.09	junenol	C25	4547.44	2-borneol	C4	4197.71	(−)-verbenone	C9	3884.48
*γ*-pironene	C53	3777.84	*trans*-4-thujanol	C31	4372.10	caryophyllene oxide	C9	4072.41	neointermedeol	C36	3875.57
eucalyptol	C31	3512.37	*cis*-carveol	C16	4218.90	mustakone	C16	3428.25	ledane	C32	3635.38
copaene	C28	3422.12	2-ethylidene-6-methyl-3,5-heptadienal	C43	4017.18	*β*-selinene	C23	3130.88	isoaromadendrene epoxide	C28	3631.91
camphor	C8	3298.98	caryophyllene oxide	C14	3864.40	(−)-xanthorrhizol	C27	2908.70	2-borneol	C12	3330.03
methyl isocostate	C36	3249.50	*α*-himachalene	C51	3523.03	*β*-costol	C22	2801.19	(+)-*α*-cyperone	C6	3159.83
*γ*-costol	C51	3240.85	*γ*-pironene	C37	3487.45	himbaccol	C12	2651.97	(−)-calamenene	C1	2998.45
caryophyllene oxide	C21	3158.27	neointermedeol	C27	3457.32	ledol	C15	2651.97	*δ*-cadinene	C49	2985.95
4-thujanol	C12	2986.18	eucalyptol	C21	3336.46	palustrol	C18	2397.18	*cis*-carveol	C22	2899.12
*trans*-4-thujanol	C44	2986.18	bornyl acetate	C45	3168.03	4(15),5,10(14)-germacratrien-1-ol	C11	2333.46	linalool	C34	2869.81
(−)-*β*-bourbonene	C4	2968.32	*γ*-terpinene	C38	3009.81	isoledene	C13	2331.30	mustakone	C35	2747.32
2-borneol	C10	2951.02	chrysanthenone	C46	2935.24	*α*-gurjunene	C19	2318.41	*α*-himachalene	C42	2652.59
camphene	C17	2884.04	1-(1-butynyl) cyclopentanol	C41	2756.51	(±)-*α*-curcumene	C3	2280.32	neoisolongifolene	C23	2606.73
*cis*-*p*-menth-2-en-1-ol	C27	2594.42	copaene	C19	2752.69	*τ*-muurolol	C25	1888.10	*β*-selinene	C47	2491.54
(±)-piperitone	C6	2490.96	*cis*-*p*-menth-2-en-1-ol	C18	2519.15	*α*-neocallitropsene	C20	1815.24	*α*-copaene	C40	2418.91
(−)-carvone	C2	2489.47	(−)-*β*-bourbonene	C3	2434.89	*α*-muurolene	C38	1762.92	himbaccol	C27	2395.18
selina-4,11-dien	C42	2458.10	yogomi alcohol	C32	2385.97				ledol	C33	2395.18
(+)-*δ*-cadinene	C11	2187.37	selina-4,11-dien	C29	2376.64				camphor	C31	2272.53

^a^ Numbering of compounds in the R cluster analysis diagram (Figure S3). ^b^ Scores of components from R cluster analysis.

**Table 3:** Proteins are included in the top 50 of the score by R cluster analysis.

GAAEOs	NO. ^c^	Score ^d^	LAAEOs	NO. ^c^	Score ^d^	GAVEOs	NO. ^c^	Score ^d^	LAVEOs	NO. ^c^	Score ^d^
NFKB1	T26	14,010.38	NFKB1	T26	14,260.94	NFKB1	T35	13,462.24	NFKB1	T40	13,974.02
PIK3CD	T118	9959.54	MAPK1	T70	10,134.16	MAPK1	T171	10,188.79	MAPK1	T139	10,667.82
MAPK1	T70	9895.40	PIK3CD	T105	9055.98	PIK3CD	T161	6665.46	PIK3CD	T110	6385.01
PRKCA	T175	6076.31	PIK3CA	T134	6661.89	PRKCA	T120	6410.28	PIK3CA	T260	4726.91
PIK3CA	T144	5979.37	PRKCA	T150	5812.37	PIK3CA	T180	5693.44	PIK3CB	T261	4431.85
PIK3R1	T156	5327.63	PIK3CB	T263	5567.77	PIK3CB	T181	5693.44	PIK3R1	T186	4424.04
RELA	T215	5264.89	RELA	T190	5363.08	RELA	T46	4034.71	NOS2	T42	4065.17
PIK3CB	T281	5001.83	NOS2	T47	4740.83	PIK3R1	T144	3792.55	PRKCA	T197	3704.80
NOS2	T47	4484.77	PIK3R1	T201	3986.61	NOS2	T36	3742.43	RELA	T120	3315.37
PRKCB	T99	3924.41	PRKCB	T245	3899.08	CASP9	T57	2926.28	PRKCB	T164	3205.22
CASP9	T137	3217.58	CASP9	T127	3476.93	ITGB3	T219	2724.65	CASP9	T58	2908.34
NOS3	T27	2945.04	NOS3	T27	3296.31	RAC1	T266	2605.33	NOS3	T43	2799.68
STAT3	T125	2873.77	IDH1	T286	2816.61	ADAM10	T178	2482.65	ACACA	T132	2548.51
SLC9A1	T122	2457.80	CYP1A2	T116	2592.61	NOS3	T37	2260.63	ITGB1	T135	2498.96
TP53	T230	2279.98	ACACA	T63	2570.09	PLA2G1B	T94	2167.69	FBP1	T283	2494.99
CYP1A2	T86	2186.07	PLA2G1B	T144	2496.26	MAOA	T32	2087.59	PRKCG	T166	2431.40
GUSB	T245	2182.93	ENPP1	T271	2386.76	MAPK14	T6	1975.62	STAT3	T86	2402.65
MTOR	T201	2178.04	TP53	T197	2281.28	CYP1A2	T68	1920.10	UGT2B7	T255	2334.90
ENPP1	T290	2166.02	MAOA	T66	2247.70	MTOR	T158	1832.80	CYP1A2	T90	2251.76
						STAT3	T126	1802.37	TP53	T239	2194.28
						UGT2B7	T172	1774.58	PLA2G1B	T76	2169.10
						SLC9A1	T51	1773.40			

^c^ Numbering of targets in the R cluster analysis diagram (Figure S3). ^d^ Scores of targets from R cluster analysis.

## Data Availability

Not applicable.

## Supplementary Materials

The following supporting information can be downloaded at: https://www.mdpi.com/article/10.3390/molecules28093927/s1, Tables S1–S3, Figures S1–S5.

## Sample Availability

Samples of the compounds are available from the authors.
