# Supplementary material for: Comparative Analysis of Active Ingredients and Potential Bioactivities of Essential Oils from Artemisia argyi and A. verlotorum
Source: Molecules. 2023 May 6;28(9):3927. doi: 10.3390/molecules28093927 (PMC10180244; doi:10.3390/molecules28093927)
Supplement: Supplementary file 1 [file molecules-28-03927-s001.zip › molecules-2344534-supplementary.pdf]

SUPPLEMENTARY MATERIAL

Comparative analysis of active ingredients and potential bioactivities of essential oils from *Artemisia argyi* and *A. verlotorum*

Yun-Fen Wang, [a,d] Yang Zheng, [b,d] Yang Feng, [a] Hao Chen, [a] Shao-Xing Dai, [b] Yifei Wang, [c, *] and Min Xu [a, *]

[a] Center for Pharmaceutical Sciences, Faculty of Life Science and Technology, Kunming University of Science and Technology, Chenggong Campus, Kunming 650500, China

[b] State Key Laboratory of Primate Biomedical Research; Institute of Primate Translational Medicine, Kunming University of Science and Technology, Kunming, Yunnan, 650500, China

[c] Guangzhou Jinan Biomedicine Research and Development Center, Institute of Biomedicine, College of Life Science and Technology, Jinan University, Guangzhou, 510632, China

[d] These authors contributed equally to this work.

[*]Correspondence: xumin@kust.edu.cn (M.X.); twang-yf@163.com (Y.W.);

Tel.: +86-0871-65920253

Supplementary material

	Content	Page
Table S1	Composition of the EOs from *Artemisia.*	3
Table S2	Common components of EOs.	7
Table S3	Numbering correspondence table for Figure 4.	9
Figure S1	The Yields of EOs of *Artemisia.*	10
Figure S2	Ingredient-Target-pathway network of EOs.	11
Figure S3	R cluster analysis.	15
Figure S4	Gene Ontology (GO) analysis.	16
Figure S5	Total Ions Chromatograms (TIC) of EOs from *Artemisia.*	20

Table S1. Composition of the EOs from *Artemisia.*

Compound name	Content（%）				Mass	RI(exp)	RI(lit)	ID [b]
	YP-1[a]	YP-2[a]	YP-3[a]	YP-4[a]				
neointermedeol	9.69	8.02	4.72	2.79	222.2	1629		MS
caryophyllene	8.73	9.24	1.02	3.8	204.2	1396	1416	RI, MS
caryophyllene oxide	5.93	3.42	17.06	23.7	220.2	1557	1579	RI, MS
cis-chrysanthenol	5.92				152.2	1145	1161	RI, MS
eucalyptol	5.77	11.51			154.1	1013	1030	RI, MS
(-)-4-terpinenol	5.47	5.68			154.2	1159	1182	RI, MS
2-borneol	4.54	10.09	0.52	2.86	154.2	1147	1165	RI, MS
α-terpineol	3.21	3.23			154.2	1172	1187	RI, MS
(-)-*cis*-verbenol	2.81				152.2	1125	1140	RI, MS
caryophylladienol I	2.42	1.15	3.16	2.42	220.4	1607	1640	RI, MS
cis-carveol	2.35	3.06		0.12	152.2	1199	1226	RI, MS
germacrene D	1.7	1.98			204.2	1456	1478	RI, MS
spainulenol	1.58	1.06			220.4	1552	1574	RI, MS
cis-p-menth-2-en-1-ol	1.49	2.08			154.3	1111	1136	RI, MS
(*E*)-*β*-famesene	1.46				204.4	1432	1455	RI, MS
artemisia alcohol	1.38	2.63			154.2	1065	1083	RI, MS
cis-piperitol	1.33	1.4			154.2	1187	1193	RI, MS
chamazulene	1.25	0.53			184.3	1700		MS
humulene	0.97	0.97	0.36	5.87	204.4	1428	1451	RI, MS
β-selinene	0.95	0.82	1.15	1.36	204.4	1461	1483	RI, MS
trans-4-thujanol	0.83	1.45			154.2	1079	1065	RI, MS
γ-terpinene	0.72	0.81			136.2	1040	1057	RI, MS
4-thujanol	0.72				154.3	1048	1068	RI, MS
n-hexadecanoic acid	0.69		1.78		256.4	1933		MS
4(15),5,10(14)-Germacratrien-1-ol	0.68	0.3	1.07	0.73	220.4	1658		MS
phytol	0.57	0.12	1.46	0.31	220.4	2077		RI, MS
(±)-phytone	0.51	0.2	3.34	1.8	268.5	1812		RI, MS
selina-4,11-diene	0.49	0.3			218.3	1450	1492	RI, MS
eugenol	0.45	0.84			164.2	1335	1354	RI, MS
bicyclogermacren	0.43	0.45			204.4	1471	1491	RI, MS
copaene	0.4	0.39			204.4	1352	1375	RI, MS
eremophylene	0.39	0.19			204.4	1564	1507	MS
τ-cadinol	0.34	0.23			222.4	1613	1638	RI, MS
yogomi alcohol	0.33	0.97			154.3	983	1000	RI, MS
γ-pironene	0.31	0.36			136.2	1313	1338	RI, MS
(+)-δ-cadinene	0.3	0.25			204.4	1497	1469	RI, MS
carveol	0.28	0.3			152.2	1209	1200	RI, MS
hexadecanoic acid, methyl ester	0.28				270.5	1893		RI, MS
junenol	0.26	0.2			222.4	1589	1606	RI, MS

(±)-piperitone	0.26	0.21			152.2	1232	1250	RI, MS
perillaldehyde	0.25	0.33			152.2	1252	1268	RI, MS
alloaromadendrene	0.24				204.4	1575	1463	RI, MS
nerolidol	0.23				222.4	1537	1560	RI, MS
(-)-carvone	0.23	0.3			150.2	1222	1190	RI, MS
Camphor	0.21				152.2	1263	1277	RI, MS
β-copaene	0.2	0.27			204.4	1403	1429	RI, MS
1-octen-3-ol	0.2	0.34			128.2	963	978	RI, MS
methyl isocostate	0.2				248.4	1754		MS
9,12-octadecadienoic acid, methyl ester	0.18				294.5	2059		MS
α-terpinene	0.18	0.4			136.2	999	1015	RI, MS
linolenic acid, methyl ester	0.18				292.5	2065		MS
o-cymene	0.17				134.2	1007	1020	RI, MS
(-)-*β*-bourbonene	0.17	0.12			204.4	1360	1381	RI, MS
methyleugenol	0.17	0.21			178.2	1382	1400	RI, MS
γ-costol	0.17				220.4	1718		MS
camphene	0.13				136.2	1570	954	RI, MS
cis-chrysanthenol acetate	0.13				194.3	1239	1260	RI, MS
α-bergamotene	0.13			0.84	204.4	1410	1431	RI, MS
epi-*β*-caryophyllene	0.13		0.2	0.53	204.4	1637		MS
bornyl isovalerate	0.12				238.4	1491	1468	RI, MS
(±)-filifolone	0.12	0.28			150.2	1084	1107	RI, MS
γ-muurolene	0.11		1.15		204.4	1488	1474	RI, MS
geranyl-*α*-terpinene	0.11				272.5	1919		MS
sclareol oxide	0.11				262.4	1849		MS
(1*α*,3*α*,5*α*)-1,5-diethenyl-3-methyl-2-methylene-cyclohexane	0.1				216.2	1676		MS
β-costol		0.21	0.76	0.21	220.4	1737		MS
bornyl acetate		0.28		0.16	196.3	1263	1282	RI, MS
isogermacrene D		0.12			218.4	1418	1444	RI, MS
p-cymene		0.41			134.2	1006	1023	RI, MS
isoborneol, isovalerate		0.1			154.3	1490	1520	RI, MS
cis-*β*-farnesene		0.86	0.42	0.83	204.4	1431		MS
(+)-2-bornanone		5.14			152.2	1124	1132	RI, MS
2,3-dehydro-1,8-cineole		0.28			152.2	971	989	RI, MS
2-ethylidene-6-methyl-3,5-heptadienal		0.16			150.2	1375	1126	MS
1-(1-butynyl) cyclopentanol		0.37			138.2	1167		MS
γ-cadinene		0.14	0.66	0.59	204.2	1618		MS
β-elemene		0.11		0.4	204.4	1367	1390	RI, MS
β-bisabolene		0.1	0.35	0.46	204.4	1537	1511	RI, MS
trimethylenenorbornane		0.19			136.2	1135		RI, MS

chrysanthenone	0.35			150.2	1373	1127	RI, MS
alloaromadendrene oxide	0.23	0.5		220.4	1593	1462	RI, MS
α-himachalene	0.15		0.14	204.4	1572	954	RI, MS
ledane			0.32	204.4	1512	1497	RI, MS
palustrol		0.97	1.57	222.4	1540	1566	RI, MS
ledol		2.52	2.69	222.2	1575	1568	RI, MS
himbaccol		3.56	3.59	222.4	1565	1589	RI, MS
mustakone		1.84	1.1	218.3	1650		RI, MS
(+)-*α*-cyperone			0.74	218.3	1593	1739	RI, MS
(±)-*α*-curcumene		1.87	2.64	202.3	1457	1480	RI, MS
α-gurjunene		3.82	0.21	204.4	1384	1407	RI, MS
(+)-*α*-calacorene		0.56	0.86	200.3	1516	1539	RI, MS
(-)-*α*-muurolene			0.37	204.4	1474	1496	RI, MS
neoisolongifolene			2.75	220.4	1630		MS
aromandendrene		0.1	0.83	204.4	1636		MS
α-copaene			0.26	204.4	1351	1375	RI, MS
δ-cadinene			3.55	204.4	1497	1511	RI, MS
1,5-cadinadiene			0.44	204.4	1618	1495	RI, MS
eudesma-4,11-diene			0.63	204.4	1449		RI, MS
β-calacorene		0.19	0.47	200.3	1535	1560	RI, MS
isoledene		0.15	0.27	204.4	1466	1377	RI, MS
(-)-calamenene			0.53	202.3	1380	1518	RI, MS
α-neocallitropsene		1.04	0.48	206.4	1654		MS
cis-2,2-dimethyl-3-(3-methyl-2-butenyl)-6-methylene cyclohexanemethanol			0.36	158.2	1667		MS
(+)-*β*-eudesmol		6.67	3.52	222.4	1622		MS
isospathulenol		3.77	2.5	220.4	1600	1630	RI, MS
caryophylladienol II		1.25	1.06	220.4	1603	1633	RI, MS
τ-muurolol		0.17	1.04	222.4	1613	1640	RI, MS
isoaromadendrene epoxide			0.27	220.4	1662		MS
terpinen-4-ol			0.21	154.3	1156	1175	RI, MS
(-)-myrtenol			0.26	152.2	1174	1213	RI, MS
(-)-*trans*-pinocarveol			0.29	152.2	1117	1137	RI, MS
linalool			0.1	154.3	1081	1097	RI, MS
l-camphor			0.27	152.2	1122	1139	RI, MS
(1*S*)-(-)-verbenone			0.12	150.2	1187	1204	RI, MS
4-methylene-5-hexenal			0.1	110.2	1085	897	RI, MS
2,5-dimethyl-3-methylene-1,5-heptadiene	0.31			136.2	1591	915	RI, MS
9-(1-methylethylidene)-1,5-cycloundecadiene			0.31	204.4	1736		MS
(-)-xanthorrhizol		0.63		218.3	1723		MS

1,1,7,7a-tetramethyl-1a,2,6,7,7a,7b-hexahydro-1*H*-cyclopropa[a]-naphthalene			0.21		202.3	1761		MS
α-muurolene			0.2		204.4	1420	1440	RI, MS
trans-*α*-bergamotene			0.6		204.4	1469	1438	RI, MS
8,9-dehydro-neoisolongifolene			0.17		202.3	1795		MS
1-ethenyl-1-methyl-2,4-bis(1-methylethenyl)-cyclohexane			0.14		204.4	1367	1389	RI, MS
valerena-4,7(11)-diene			0.16		204.4	1756		MS
(-)-isocaryophyllene			0.37		204.4	1689		MS
pentamethylcyclopentadiene			0.53		136.2	1726		MS
isophytol			0.11		296.3	1913		MS
manool oxide			0.15		290.5	1978		MS
m-anisalcohol			3.36		138.2	1180	1198	RI, MS
chlorpyrifos			0.42		350.6	1957		MS
4-methylene-6-(1-propenylidene)-cyclooctene			0.11		160.3	1825		MS
**monoterpenoids**	**38.71**	**31.29**	**1.05**	**4.4**				
**sesquiterpenoids**	**38.42**	**52.01**	**63.56**	**77.72**				
**diterpenoids**	**0.11**		**0.26**					
**aromatics**	**2.03**	**1.58**	**3.36**					
**aliphatics**	**3.11**	**1.03**	**6.68**	**2.83**				
**others**			**0.42**					
**total**	**82.38**	**85.91**	**75.32**	**84.95**				

[a] Percent calculated from MS data.; [b] Identification method: MS, identified on the basis of computer matching of the mass spectra with those of the Wiley and Mass Finder libraries and comparison with literature data.

Table S2. Common components of EOs.

Common components of GAAEOs and LAAEOs	Common components of GAVEOs and LAVEOs	Common components of GAVEOs and LAVEOs
(-)-4-terpinenol	(+)-*α*-calacorene	neointermedeol
(-)-carvone	(+)-*β*-eudesmol	caryophyllene
(-)-*β*-bourbonene	(±)-phytone	caryophyllene oxide
(±)-filifolone	(±)-*α*-curcumene	2-borneol
(±)-phytone	2-borneol	caryophylladienol I
(±)-piperitone	aromandendrene	humulene
1-octen-3-ol	caryophylladienol I	*β*-selinene
2-borneol	caryophylladienol II	4(15),5,10(14)-Germacratrien-1-Ol
(+)-*δ*-cadinene	caryophyllene	phytol
artemisia alcohol	caryophyllene oxide	(±)-phytone
bicyclogermacren	*cis*-*β*-farnesene	
carveol	epi-*β*-caryophyllene	
caryophylladienol I	4(15),5,10(14)-Germacratrien-1-Ol	
caryophyllene	himbaccol	
caryophyllene oxide	humulene	
chamazulene	isoledene	
cis-carveol	isospathulenol	
cis-piperitol	ledol	
cis-p-menth-2-en-1-ol	mustakone	
copaene	neointermedeol	
eremophylene	palustrol	
eucalyptol	phytol	
eugenol	*α*-gurjunene	
4(15),5,10(14)-Germacratrien-1-Ol	*α*-neocallitropsene	
germacrene D	*β*-bisabolene	
humulene	*β*-calacorene	
junenol	*β*-costol	
methyleugenol	*β*-selinene	
neointermedeol	*γ*-cadinene	
perillaldehyde	*τ*-muurolol	
phytol		
selina-4,11-dien		
spainulenol		

trans-4-thujanol
yogomi alcoho
α-terpinene
α-terpineol
β-copaene
β-selinene
γ-pironene
γ-terpinene
τ-cadinol

Table S3. Numbering correspondence table for Figure 4.

NO.	compounds	NO.	compounds	NO.	targets	NO.	pathway
C1	*γ*-pironene	C15	β-calacorene	T1	NTRK3	P1	PI3K-Akt signaling pathway
C2	o-cymene	C16	pentamethylcyclopentadiene	T2	NOS2	P2	Pathways of neurodegeneration - multiple diseases
C3	neointermedeol	C17	m-anisalcohol	T3	NFKB1	P3	Pathways in cancer
C4	methyleugenol	C18	himbaccol	T4	CNR2	P4	Neuroactive ligand-receptor interaction
C5	junenol	C19	caryophyllene oxide	T5	CHRM4	P5	MicroRNAs in cancer
C6	eugenol	C20	alloaromadendrene oxide	T6	AR	P6	Metabolic pathways
C7	eucalyptol	C21	2-borneol	T7	APOBEC3A	P7	Chemical carcinogenesis-receptor activation
C8	*cis*-carveol	C22	1-ethenyl-1-methyl-2,4-bis(1-methylethenyl)-cyclohexane	T8	APEX1	P8	cAMP signaling pathway
C9	chamazulene	C23	α-himachalene	T9	ADRA2C	P9	Calcium signaling pathway
C10	1-octen-3-ol	C24	bornyl acetate	T10	ACHE	P10	Alzheimer disease
C11	β-elemene	C25	9-(1-methylethylidene)-1,5-cycloundecadiene	T11	HTR1E		
C12	trimethylenenorbornane	C26	(1S)-(-)-verbenone	T12	NR3C2		
C13	p-cymene			T13	KDM1A		
C14	β-selinene			T14	GPR55		

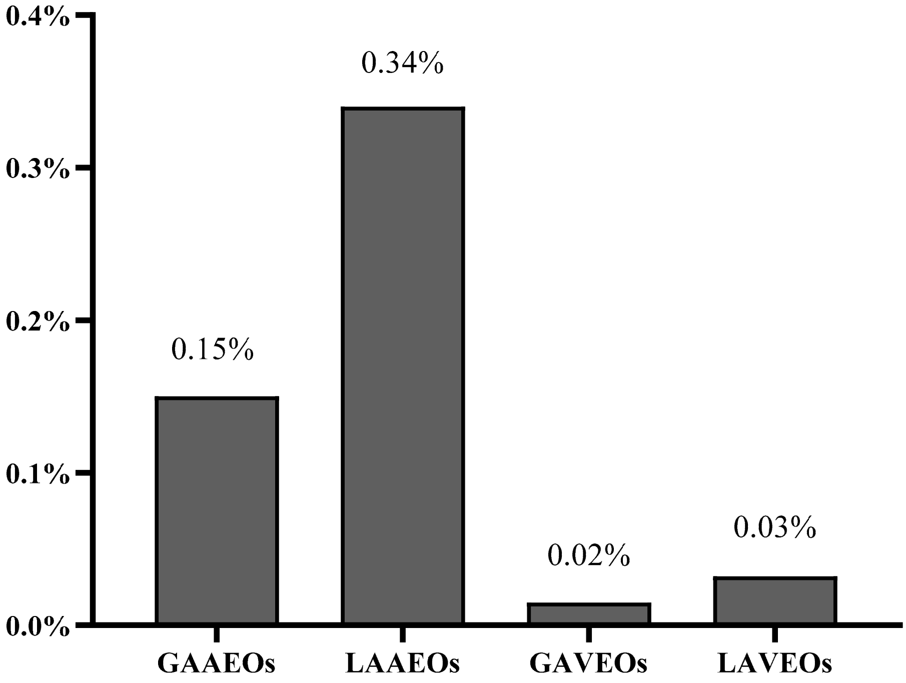

Figure S1. The Yields of EOs of *Artemisia*.

GAAEOs

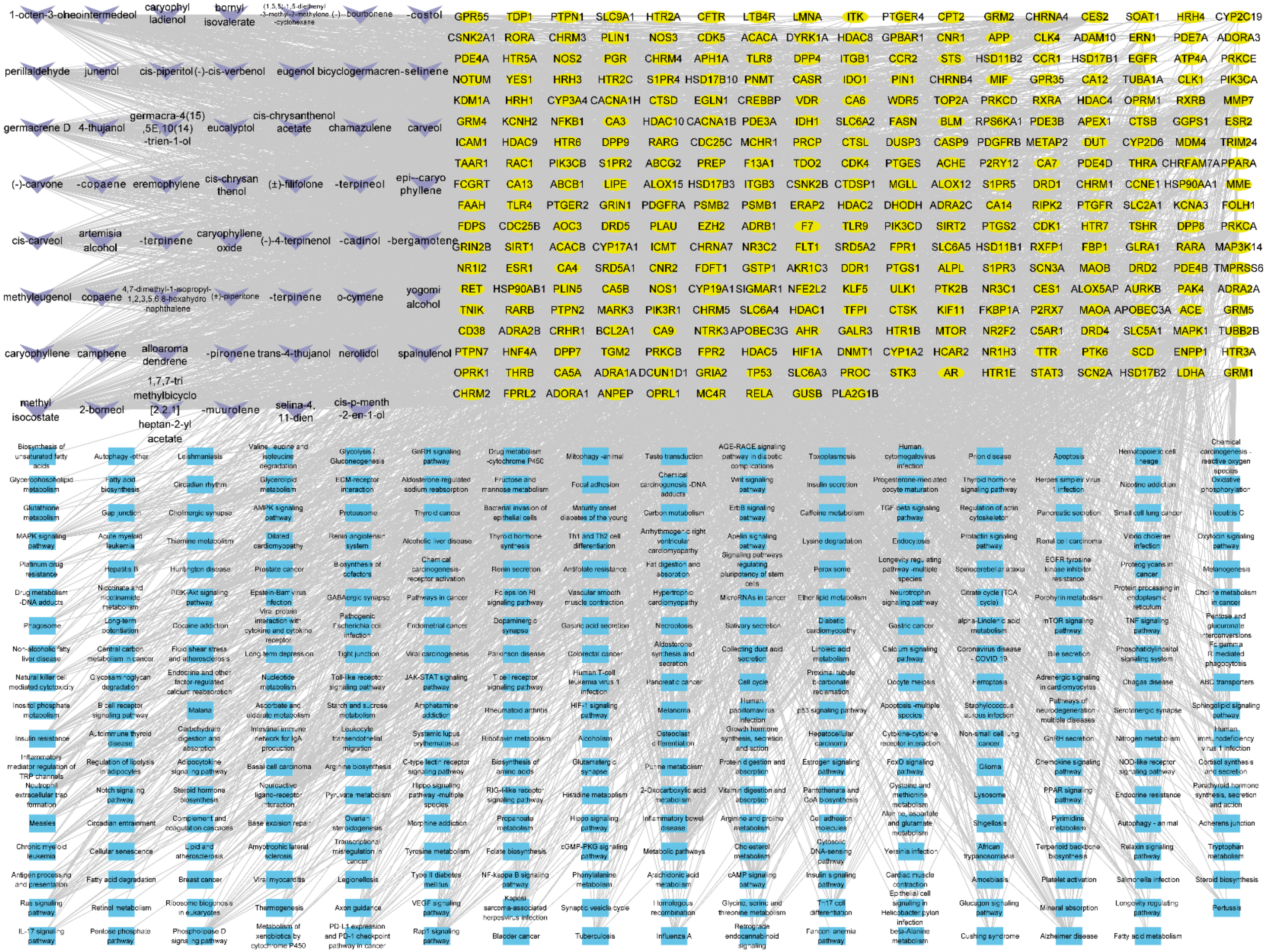

LAAEOs

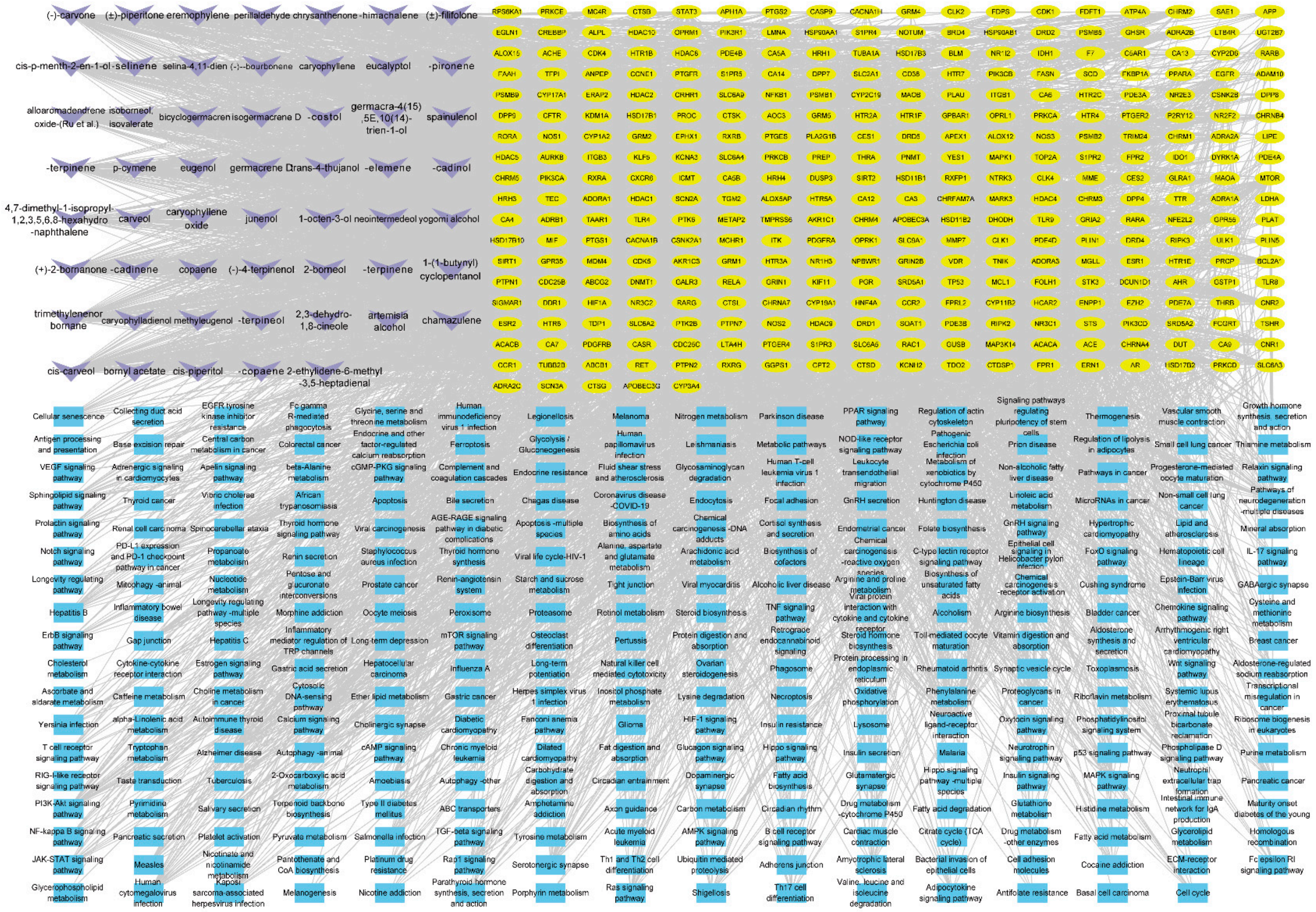
(-)-carvone
(±)-piperitone
eremophylene
perillaldehyde
chrysanthenone
-himachalene
(±)-filifolone
cis-p-menth-2-en-1-ol
-selinene
selina-4,11-dien
(-)--bourbonene
caryophyllene
eucalyptol
-pironene
alloaromadendrene oxide-(Ru et al.)
isoborneol, isovalerate
bicyclogermacren
isogermacrene D
-costol
germacra-4(15),5E,10(14)-trien-1-ol
spainulenol
-terpinene
p-cymene
eugenol
germacrene D
trans-4-thujanol
-elemene
-cadinol
4,7-dimethyl-1-isopropyl-1,2,3,5,6,8-hexahydro-naphthalene
carveol
caryophyllene oxide
junenol
1-octen-3-ol
neointermedeol
yogomi alcohol
(+)-2-bornanone
-cadinene
copaene
(-)-4-terpinenol
2-borneol
-terpinene
1-(1-butynyl) cyclopentanol
trimethylenenor bornane
caryophylladienol
methyleugenol
-terpineol
2,3-dehydro-1,8-cineole
artemisia alcohol
chamazulene
cis-carveol
bornyl acetate
cis-piperitol
-copaene
2-ethylidene-6-methyl-3,5-heptadienal

GAVEOs

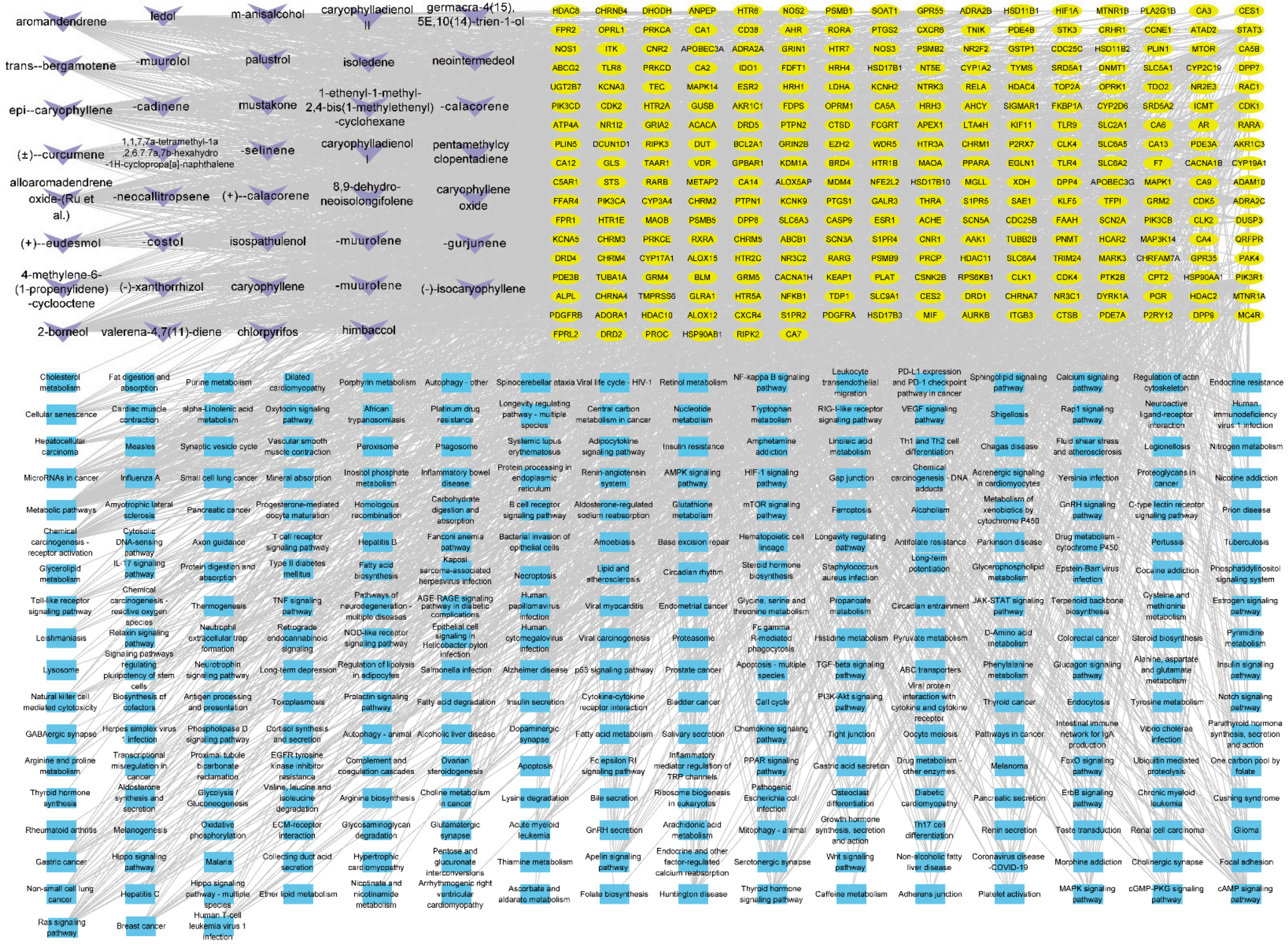

aromandendrene
ledol
m-anisalcohol
caryophylladienol II
germacra-4(15),5E,10(14)-trien-1-ol
trans--bergamotene
-muurolol
palustrol
isoledene
neointermedeol
epi--caryophyllene
-cadinene
mustakone
1-ethenyl-1-methyl-2,4-bis(1-methylethenyl)-cyclohexane
-calacorene
(±)--curcumene
1,1,7,7a-tetramethyl-1a,2,6,7,7a,7b-hexahydro-1H-cyclopropa[a]-naphthalene
-selinene
caryophylladienol I
pentamethylcyclopentadiene
alloaromadendrene oxide-(Ru et al.)
-neocallitropsene
(+)--calacorene
8,9-dehydro-neoisolongifolene
caryophyllene oxide
(+)--eudesmol
-costol
isospathulenol
-muurolene
-gurjunene
4-methylene-6-(1-propenylidene)-cyclooctene
(-)-xanthorrhizol
caryophyllene
-muurolene
(-)-isocaryophyllene
2-borneol
valerena-4,7(11)-diene
chlorpyrifos
himbaccol

LAVEOs

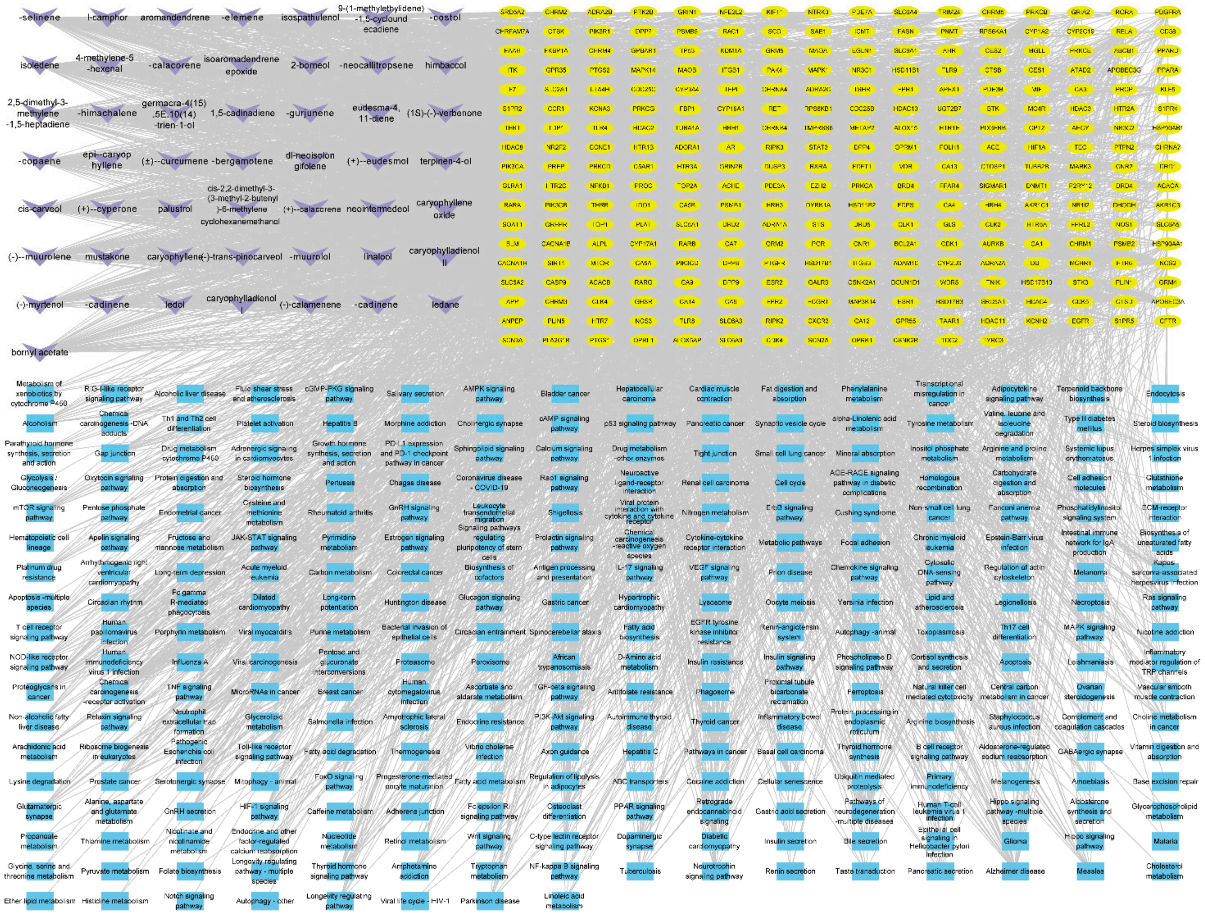

Figure S2. Ingredient-Target-pathway network of EOs. The ingredient represented by the purple V. The targets were represented by the yellow ellipse. The pathways were represented by the blue rectangle. The active ingredients were represented by the node, and the edge links the targets and the active ingredients.

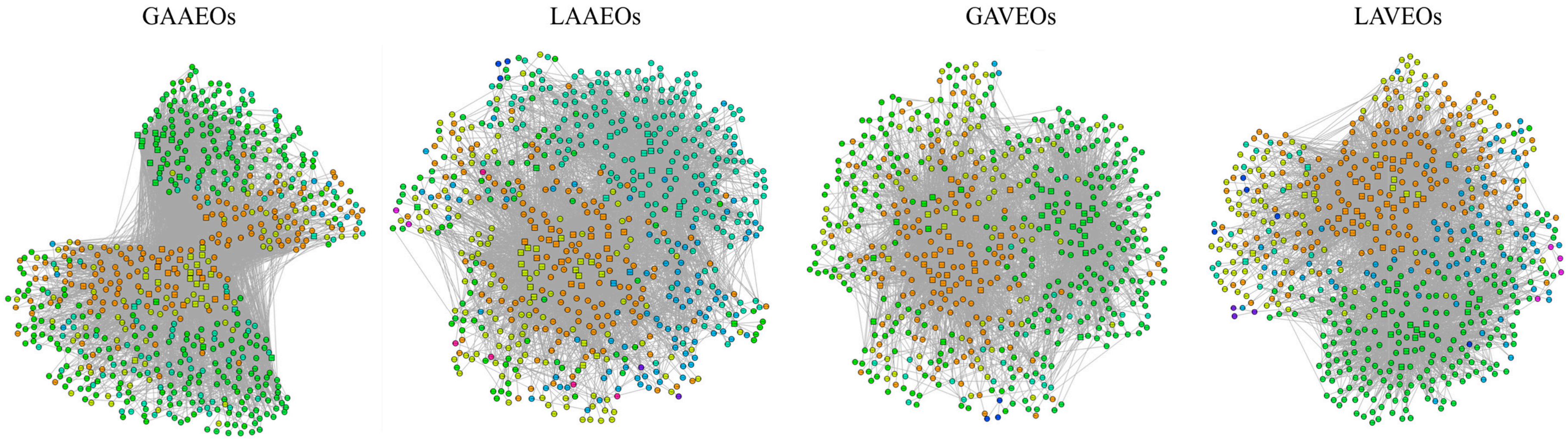

Figure S3. R cluster analysis. The related ingredients, targets and pathways information resulted in a data set which was converted to an igraph graph with “igraph” software package. Different communities were marked in different colors by a R_rainbow, and nodes with similar colors have higher associativity.

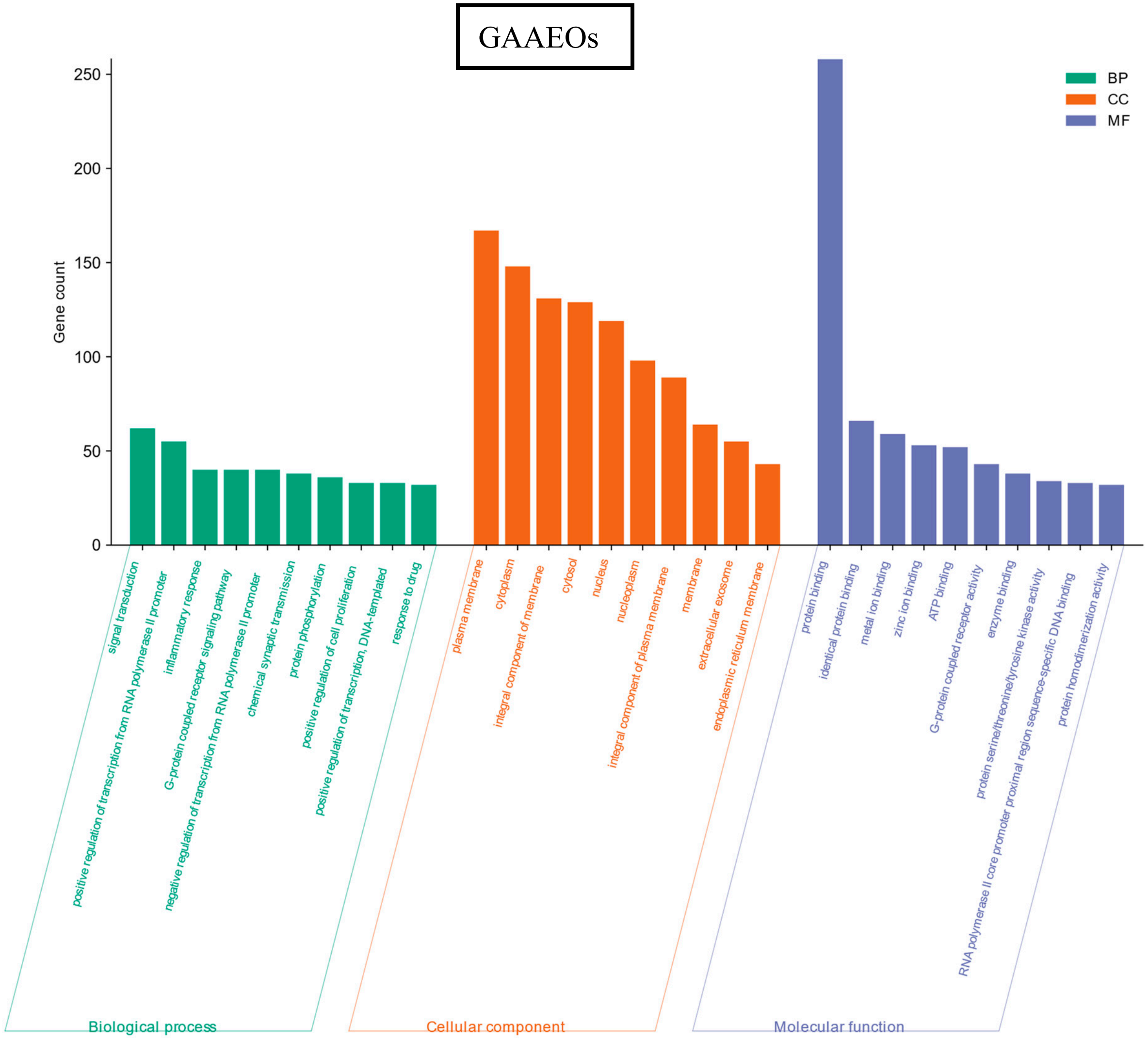
GAAEOs
Gene count
250
200
150
100
50
0
BP
CC
MF
signal transduction
positive regulation of transcription from RNA polymerase II promoter
inflammatory response
G-protein coupled receptor signaling pathway
negative regulation of transcription from RNA polymerase II promoter
chemical synaptic transmission
protein phosphorylation
positive regulation of cell proliferation
positive regulation of transcription, DNA-templated
response to drug
plasma membrane
cytoplasm
integral component of membrane
cytosol
nucleus
nucleoplasm
integral component of plasma membrane
membrane
extracellular exosome
endoplasmic reticulum membrane
protein binding
identical protein binding
metal ion binding
zinc ion binding
ATP binding
G-protein coupled receptor activity
enzyme binding
protein serine/threonine/tyrosine kinase activity
RNA polymerase II core promoter proximal region sequence-specific DNA binding
protein homodimerization activity
Biological process
Cellular component
Molecular function

LAAEOs

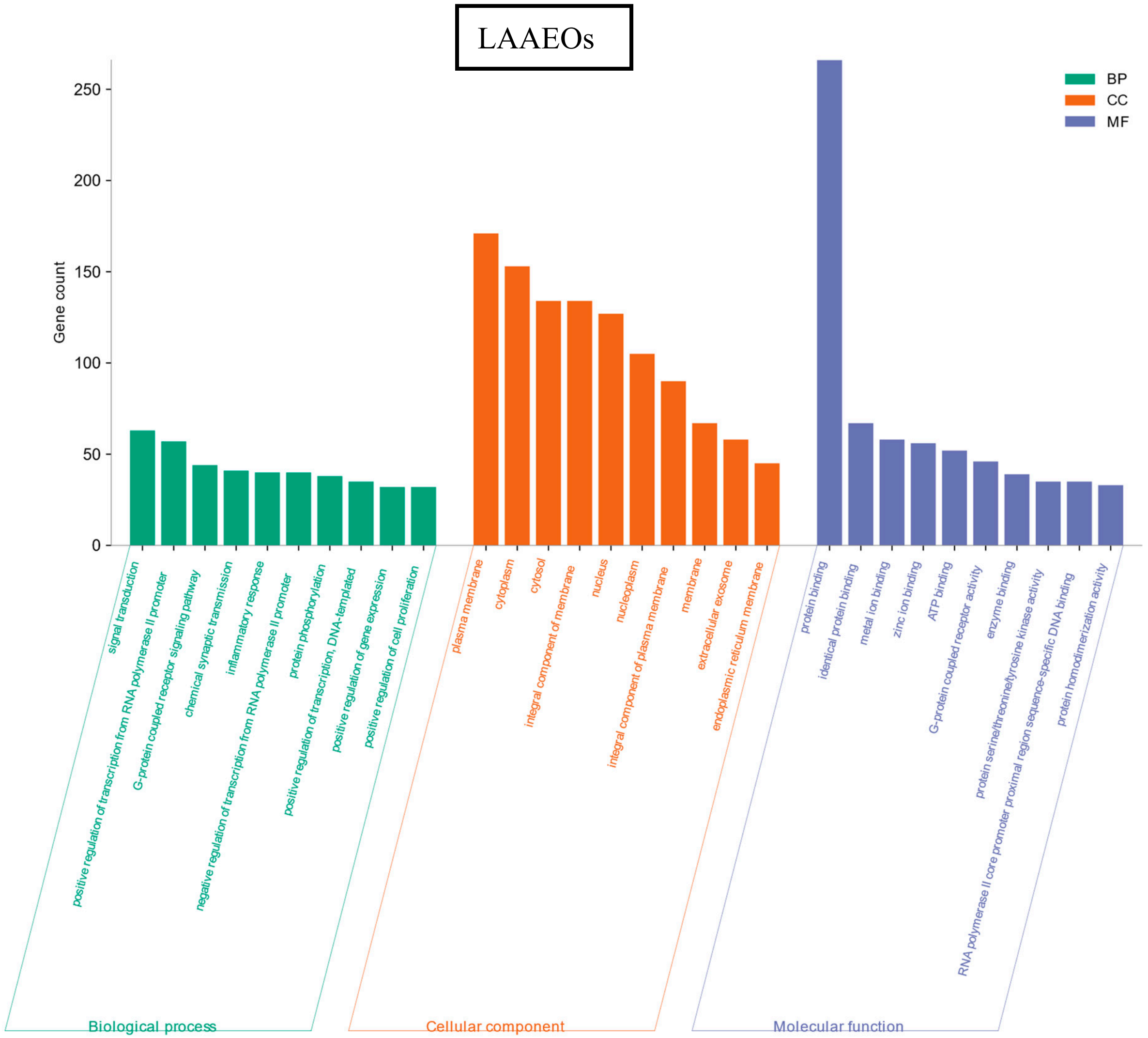
BP
CC
MF
Gene count
250
200
150
100
50
0
signal transduction
positive regulation of transcription from RNA polymerase II promoter
G-protein coupled receptor signaling pathway
chemical synaptic transmission
inflammatory response
negative regulation of transcription from RNA polymerase II promoter
protein phosphorylation
positive regulation of transcription, DNA-templated
positive regulation of gene expression
positive regulation of cell proliferation
plasma membrane
cytoplasm
cytosol
integral component of membrane
nucleus
nucleoplasm
integral component of plasma membrane
membrane
extracellular exosome
endoplasmic reticulum membrane
protein binding
identical protein binding
metal ion binding
zinc ion binding
ATP binding
G-protein coupled receptor activity
enzyme binding
protein serine/threonine/tyrosine kinase activity
RNA polymerase II core promoter proximal region sequence-specific DNA binding
protein homodimerization activity
Biological process
Cellular component
Molecular function

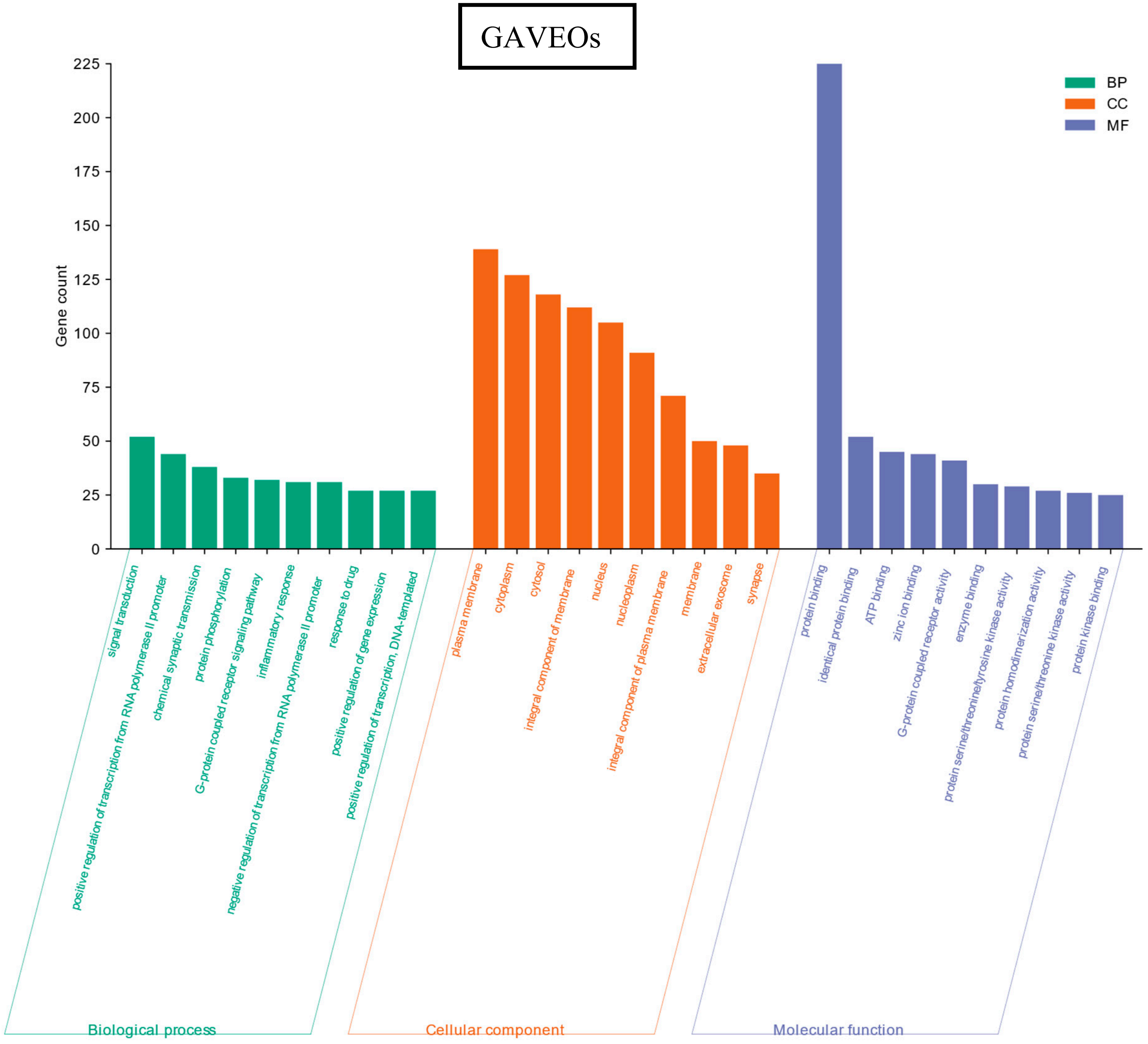
GAVEOs
Gene count
225
200
175
150
125
100
75
50
25
0
BP
CC
MF
signal transduction
positive regulation of transcription from RNA polymerase II promoter
chemical synaptic transmission
protein phosphorylation
G-protein coupled receptor signaling pathway
inflammatory response
negative regulation of transcription from RNA polymerase II promoter
response to drug
positive regulation of gene expression
positive regulation of transcription, DNA-templated
plasma membrane
cytoplasm
cytosol
integral component of membrane
nucleus
nucleoplasm
integral component of plasma membrane
membrane
extracellular exosome
synapse
protein binding
identical protein binding
ATP binding
zinc ion binding
G-protein coupled receptor activity
enzyme binding
protein serine/threonine/tyrosine kinase activity
protein homodimerization activity
protein serine/threonine kinase activity
protein kinase binding
Biological process
Cellular component
Molecular function

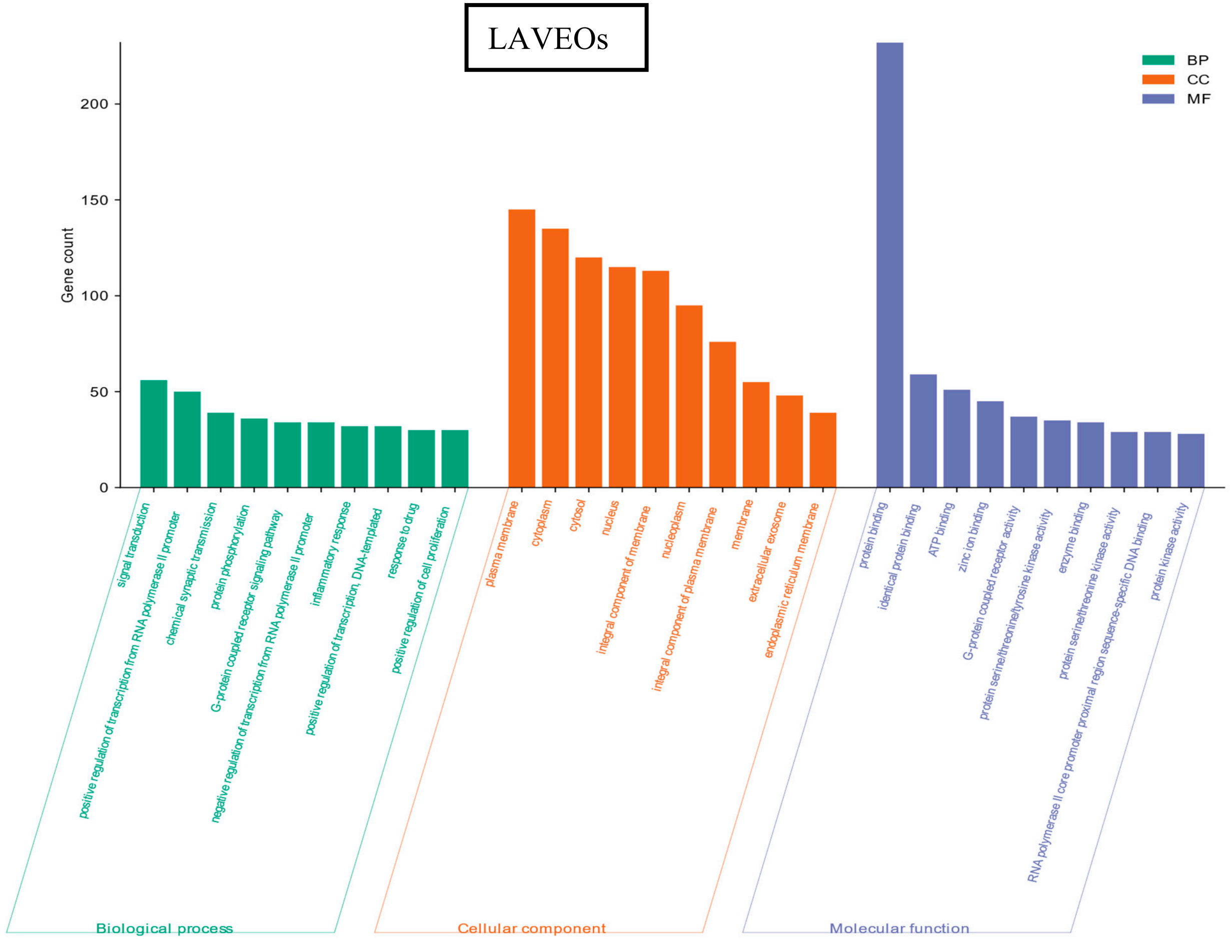

Figure S4. Gene Ontology (GO) analysis. Main Gene Ontology terms enriched by major hubs from cluster Profiler. The top 10 terms of BP, MM, and CC measured by adjusted value were selected to demonstrate.

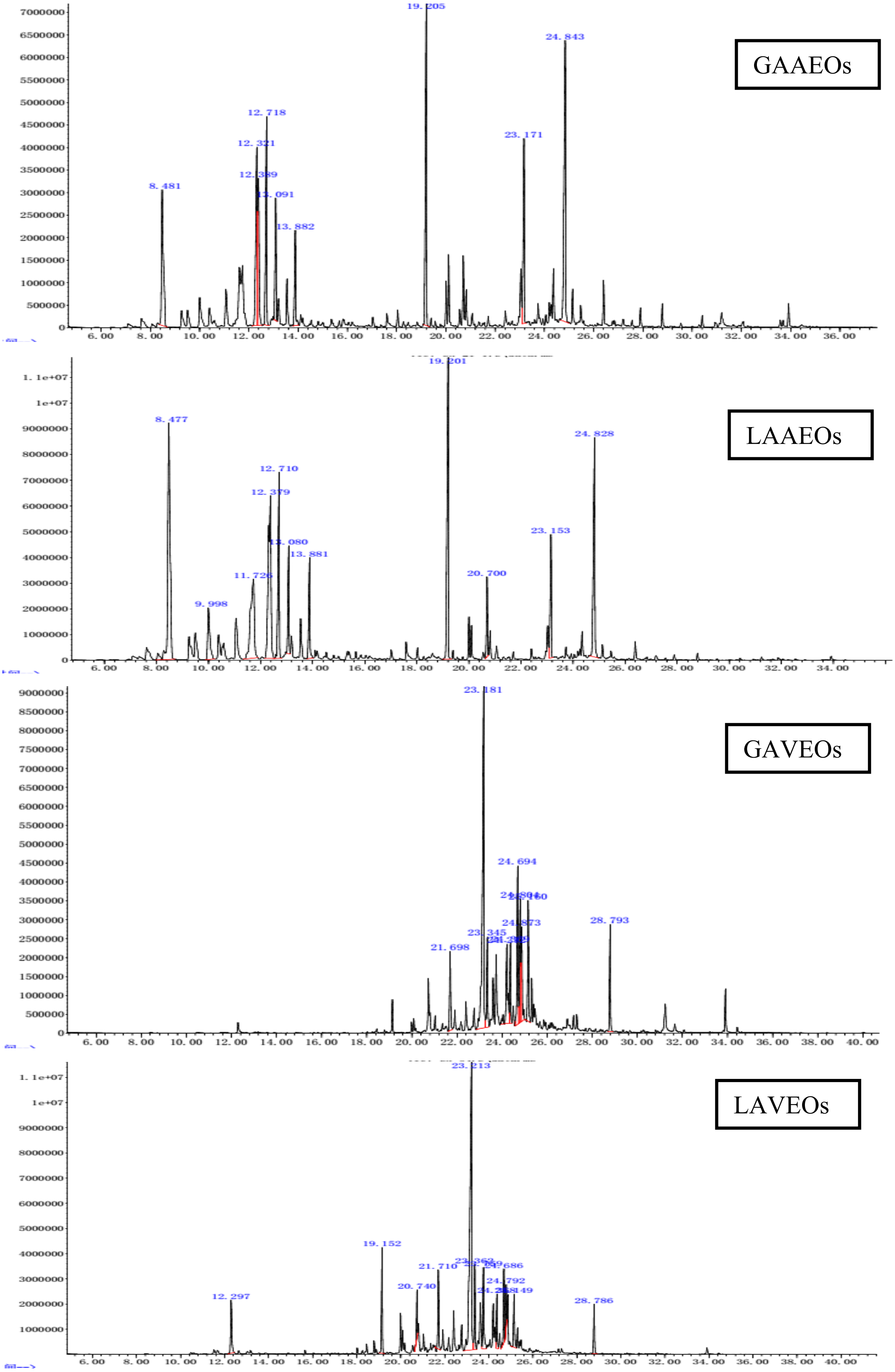

Figure S5 Total Ions Chromatograms (TIC) of EOs from *Artemisia*.
